# Transcriptomic dynamics reveals sequential acquisition of complement resistance during prolonged starvation of Trypanosoma cruzi epimastigote

**DOI:** 10.1590/0074-02760250127

**Published:** 2026-03-06

**Authors:** Leticia Pérez-Díaz, Pablo Smircich, Fabricio Hernandez, Martin Ciganda, Ma Ana Duhagon, Beatriz Garat

**Affiliations:** 1 Facultad de Ciencias, Sección Genómica Funcional, Montevideo, Uruguay; 2 Instituto de Investigaciones Biológicas Clemente Estable, Departamento de Genómica, Montevideo, Uruguay; 3 Facultad de Medicina, Departamento de Genética, Montevideo, Uruguay

**Keywords:** Trypanosoma cruzi, life cycle development, transcriptomics

## Abstract

**BACKGROUND:**

The life cycle of the parasitic protozoan *Trypanosoma cruzi*, the etiological agent of Chagas disease (CD), includes two well-recognised insect-dwelling stages: the replicative non-infective epimastigotes and the non-replicative infective metacyclic trypomastigotes. Nonetheless, the existence of multiple intermediate forms has been reported. Since nutrient restriction is considered one of the main factors driving metacyclogenesis and is very frequent due to the long-term starvation periods that the insect vectors commonly undergo, we have studied the transcriptomic effects of nutrient restriction on long-lasting epimastigote cultures. We previously reported that in these conditions, we observed a long stationary phase characterised by an RNA content per cell three times smaller than the epimastigote’s and a distinctive transcriptomic profile. Remarkably, our study identified gene expression changes that distincty characterise transitional parasite forms enriched by nutrient restriction.

**OBJECTIVES:**

In this work we focused on pathogenic genes to further characterise the transcriptomic dynamics accompanying the nutrient restriction within the insect-dwelling parasite stage.

**METHODS:**

The alterations of morphology, growth rate and complement resistance of parasite population on long-lasting epimastigote cultures as well as the transcriptomic dynamics was studied.

**FINDINGS:**

We found a gene expression early rise of surface proteins (such as trans-sialidase and GP63) and even a rise of TcTASV and δ-amastin, which is not accompanied by increased expression of metacyclic transcript markers. In addition, we found increased expression of genes coding for proteins involved in two other processes activated during the differentiation of epimastigotes to the infective form of the parasite: autophagy (Atg4, Atg7, Atg8.2) and complement resistance (TcCRP and T-DAF).

**MAIN CONCLUSIONS:**

Altogether, these results, plus our previous identification of transcriptomic markers for transitional parasites, further support earlier proposals of a specific parasite stage that morphologically resembles epimastigotes but exhibits distinctive biological characteristics, including key features related to infectivity.

## INTRODUCTION

*Trypanosoma cruzi* (Kinetoplastidae, Trypanosomatidae) is a parasitic protozoan that causes Chagas disease (CD),^1^ a major socio-economic problem in Latin America, where it is considered endemic in 21 countries, with circa 8 million people infected and about 100 million at risk of infection.^2^ The parasite is transmitted by blood-sucking triatomines widely distributed in Latin America. Since the parasite can also be transmitted by contaminated food, congenitally from mother to child and through contaminated blood or organ donations, CD has spread to non-endemic areas such as North America, Europe, and the western Pacific, due to migratory flows.^3^

As described by Chagas, the parasite has different stages along its complex life cycle.^1^ At least four stages alternating between triatomine vectors (Triatoma infestans, Hemiptera, Reduviidae), and mammalian hosts are currently accepted. The non-infective epimastigote form, which actively replicates in the vector’s midgut, differentiates into non-replicative metacyclic trypomastigotes in the hindgut. These forms are deposited with faeces and are responsible for the infection of mammals. In the mammalian hosts, the metacyclic trypomastigotes infect cells differentiating into intracellular replicative amastigotes, which finally differentiate into the blood non-replicative trypomastigotes that, after cell lysis, can invade other cells and tissues, producing the clinical manifestations of CD.

The parasite transition from epimastigotes to the infective metacyclic trypomastigote is a critical biological step in establishing the infection. This process, known as metacyclogenesis, takes place along the insect rectum, where parasites are pulled through increasingly nutrient-restricted environments. The reduced nutrient availability is the primary stimulus to induce processes such as autophagy, whose participation in the parasite differentiation process has been established.^4^ ^5^ Several morphological and structural features distinguish insect epimastigotes from metacyclic trypomastigotes. These include modifications of the relative nucleus-kinetoplast location, elongation of the cytoplasm, nucleolar disaggregation, dispersed content of heterochromatin, increase in the flagellum pocket size, and several concomitant metabolic and physiological changes.^6^ ^7^ ^8^ Metacyclic trypomastigotes also show an increased expression of proteins associated with virulence [e.g., GP63, mucins, and mucin-associated surface proteins (MASP)], and the specific metacyclic protein marker metacyclins (Met-II and Met-III).^9^ ^10^ ^11^ In contrast to metacyclic trypomastigotes, epimastigotes are highly susceptible to complement-mediated lysis^12^ though the ability to resist the complement differs among different *T. cruzi* strains.^13^ It has been early recognised that stage specific gene expression precedes morphological changes during metacyclogenesis.^14^

Despite constitutive transcription of protein-coding genes in kinetoplastids, significant transcriptomic changes between epimastigotes and metacyclic trypomastigote have been reported by us and others.^15^ ^16^ ^17^ ^18^ ^19^ ^20^ Remarkably, using traslatomics, we established translation efficiency as a crucial developmental regulatory step along this transition.^15^ Additionally, significant proteomic differences between these two developmental stages were reported.^21^ ^22^ Indeed, the analysis of in vitro cultured exponential and initial stationary phase epimastigotes has revealed either expression changes of genes related to cell cycle, pathogenesis, and metabolic processes^23^ as well as proteins related to the replication status^24^ and metabolic switch from glucose to amino acid consumption in stationary phase epimastigotes.^25^ Consistently, a recent metabolome study of nutritional and oxidative stress supports the rapid *T. cruzi* adaptation to environmental changes.^26^ While these molecular changes may reflect the beginning of the metacyclogenesis process, the existence of an intermediate distinctive preadaptive stage with the ability to differentiate either into the metacyclic form or to return to the replicative epimastigote stage depending on the availability of nutrients has been proposed.^27^ It is well recognised that the enormous amount of blood ingested and the long period of starvation affect the intestinal environments in the vector, yielding different proportions of developmental stages, including not only epimastigotes and metacyclic trypomastigotes but also many other intermediate parasite forms.^28^ Nutritional stress during the in vitro metacyclogenesis process also leads to the appearance of different intermediate parasite forms.^7^ ^29^ ^30^ ^31^ ^32^ These forms have been globally named “transitional epimastigotes”.^32^ The plasticity and complexity of *T. cruzi* forms along the life cycle spread beyond the epimastigote to metacyclic trypomastigote transition. Transient *T. cruzi* epimastigote-like forms as intermediates in the differentiation of amastigotes to trypomastigotes inside the mammalian host cells^33^ and their distinct energy and carbon source requirements compared to the other intracellular stages^34^ have been characterised. In addition, the differentiation from the trypomastigote forms (cell-derived or metacyclic) to an epimastigote-like form named “recently differentiated epimastigotes”, exhibiting complement resistance and infection ability has been recently described using cell biology and proteomic approaches.^35^

To understand the molecular changes caused by nutritional restrictions in the insect host, we have recently reported the transcriptomic changes during axenic growth of epimastigotes for more than 30 days without nutrient supplementation (prolonged starvation).^36^ In these conditions, we observed an extended stationary phase characterised by an RNA content per cell three times smaller than that of epimastigotes. This parasite population exhibited a distinctive transcriptomic profile. Ontology-enriched terms for cellular components such as contractile vacuole, reservosomes, and the mitochondria were revealed, suggesting a protagonistic role possibly related to their functions in osmoregulation and metabolic homeostasis and cell volume regulation for the adaptation to the nutrient restriction. In this parasite population, we also found a distinctive expression of genes related to DNA, granting the quiescent status in starving conditions. Remarkably, our study identified differentially expressed genes (DEGs) that constitute markers of this transitional parasite population enriched by nutrient restriction, supporting the existence of a distinctive stage between the recognised insect-dwelling forms.^27^ ^29^ ^32^

To further characterise the complex molecular dynamics accompanying the nutrient restriction within the insect branch of *T. cruzi* life cycle, we here focus on the transcriptomic analysis of pathogenic gene dynamics within the long stationary phase of axenic epimastigote culture in prolonged starvation. Firstly, we analysed the morphological characteristics of this parasite population. An increasing proportion of intermediate parasite forms with the nucleus-kinetoplast location characteristic of epimastigote and different growth resume ability was found. In addition, this parasite population exhibits an early increase of genes involved in surface protein genes, which is not accompanied by increased expression of some metacyclic transcript markers such as metacyclin II and III. Considering the involvement of surface proteins in infectivity, we focused on two other processes activated during the differentiation of epimastigotes to the infective form of the parasite: autophagy and complement resistance. We found increased expression of genes related to both these processes and the complement resistance ability was also experimentally verified. These results complement the distinctive transcriptomic profile we previously reported for transitional parasites obtained along the axenic growth of *T. cruzi* epimastigotes for over 30 days without nutrient supplementation^36^ and further support previous proposals^32^ regarding the existence of a specific parasite stage morphologically resembling epimastigotes but exhibiting distinct biological characteristics.

## MATERIALS AND METHODS

Parasite culture and morphology analyses - *T. cruzi* Dm28c strain (TcI DTU) epimastigotes were cultured as previously indicated.^36^ Three biological replicates were assessed to determine the growth curve dynamics. Parasites were directly counted by light microscopy using a Neubauer chamber, and triplicates for each independent experiment were analysed. Occasionally, parasite concentration was also verified by flow cytometry using a BD Accuri C6. Fluorescent microscopy (Leica TCS-SP5 and ZOE Fluorescent Cell Imager) was used to analyse images of 4’,6-diamidino-2-phenylindole (DAPI) of paraformaldehyde (PFA) fixed parasites. Several images were acquired, and at least 100 cells were counted in each triplicate for each independent biological replica.

Transcriptomic analysis - Expression data was obtained from.^36^ Genes were considered differentially expressed if they exhibited a log2 fold change (FC) of |1| and a false discovery rate (FDR) less than 0.05. Overrepresentation of GO terms among the differentially expressed genes was determined using the tools available at TritrypDB (http://tritrypdb.org/). A Bonferroni adjusted p-value of less than 0.5 was used as the significance cutoff. Unless stated otherwise, statistical analysis and plotting were conducted in R.

Sensibility to human serum complement - Parasites in the exponential growth phase (2 x 10^7^ parasites/mL) were diluted in culture media (1/10) containing 10% human serum that had or had not been heat treated (60ºC for 15 min). In all cases, cell viability was analysed after 24 h of incubation with the serum. The cellular viability and vitality were assessed through Propidium Iodide (PI) and Calcein-AM (CA) labelling, respectively followed by flow cytometry using a 670 nm band-pass filter (FL3) (Accuri C6, BD Bioscience). For labelling, parasites were incubated with 1x10^-6^ M CA for 1 h and 10 mg/mL PI (Thermo Fisher Scientific) for 15 min at RT in the darkness. At least three independent samples were assayed for each condition, and 10,000 events were acquired per experiment.

## RESULTS AND DISCUSSION

**Fig. 1:**
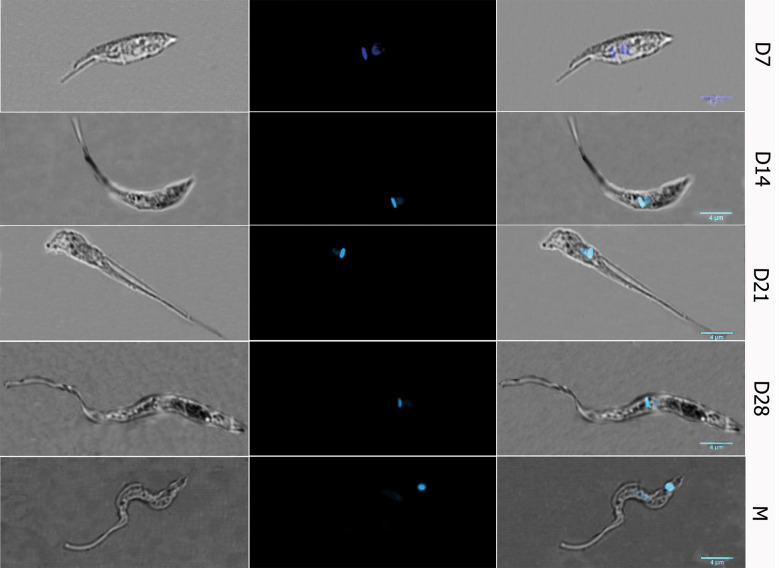
morphology of Trypanosoma cruzi epimastigotes in prolonged starvation cell culture. Representative images of parasites observed by Confocal microscopy Leica TCS-SP5 100X. Top and bottom panels: epimastigote (D7) and metacyclic trypomastigote-like parasite (M) forms observed in exponentially growing epimastigote cultures. Middle panels: intermediate forms observed in starved cell cultures at days: 14, 21 and 28 (D14, D21 and D28 respectively). Right panel: phase contrast, central panel: DAPI stained parasites, left panel: overlay of both images.

Morphology and growth kinetics during prolonged starvation of *T. cruzi* epimastigote - Along the growth curve of in vitro cultured *T. cruzi* epimastigotes for more than 30 days,^36^ the successive lag phase followed by the exponential, the stationary phase and the final death phase were observed [Supplementary data (Fig. 1A)]. The exponentially growing parasite population mostly exhibits the characteristic spindle-shaped cells and normal flagellar motility of epimastigotes (Fig. 1 top panel), although a small percentage (< 5%) of cells present a sickled-shaped morphology resembling metacyclic trypomastigotes (Fig. 1 bottom panel). Morphological differences between epimastigotes and metacyclic trypomastigotes also include the position of the kinetoplast, being anterior in the epimastigote and posterior in the metacyclic trypomastigote (compare the 2nd and 3rd columns of top and bottom panel in Fig. 1). During the stationary phase, the parasites become longer and slender, maintaining the position of the kinetoplast and flagellum base relative to the nucleus as in the epimastigote stage [Fig. 1 middle panels and Supplementary data (Fig. 1B)]. This elongation of the body and flagellum in stationary phase is well-known.^27^ ^29^ ^32^ Flagellar elongation may provide an extended nutrient uptake surface in unfavourable nutrient conditions^29^ and may constitute an early step driving the flagellar attachment required for metacyclic development.^37^ Consequently, repositioning of the kinetoplast and loss of endocytic ability have only been observed in the later stages of the metacyclogenesis process.^6^ ^7^ ^38^ However, an increasing distance between the nucleus and the kinetoplast was observed. In these conditions, we have found that the percentage of metacyclic trypomastigote within this long stationary phase displays a composed profile including a gradual increase, not surpassing 10% for a long period (3.8 ± 1.7%, 5.4± 0.4% and 7.9 ± 0.6% for the exponential phase -day 7-, early stationary phase -day 14-, and intermediate stationary phase-day 21 respectively) and then a sharp increase at the end (32.1 ± 5.4% for the final of the stationary phase -day 28-).^36^ In accordance with the distinctive capacity to resume growth of epimastigotes, metacyclic trypomastigotes, and preadaptive forms,^27^ all the analysed parasite populations were able to resume growth when the medium was replaced with fresh nutrients [Supplementary data (Fig. 1C)]. As anticipated, according to the expected doubling time of approximately one day (21.2 ± 0.7 h), parasites in the exponential growth phase are the fastest (0.79 parasites/mL.day). Afterwards, the older the parasites, the slower the growth rate.

In summary, in the assayed conditions of prolonged starvation of axenic culture of *T. cruzi* we observed the reported co-existence of multiple intermediate parasite forms.

Dynamic analysis of transcriptomic changes during prolonged starvation of *T. cruzi* epimastigote - We have previously reported that parasites in the long stationary phase provoked by prolonged starvation of epimastigote culture show a distinctive transcriptomic profile with defined DEGs markers.^36^ Nonetheless, a gradual variation of some DEGs was also noted. To study the transcriptome dynamics of the parasite population along the prolonged starvation of the axenic culture of *T. cruzi* epimastigotes, we here present the analysis of the previously reported transcriptomic data.^36^

**Fig. 2:**
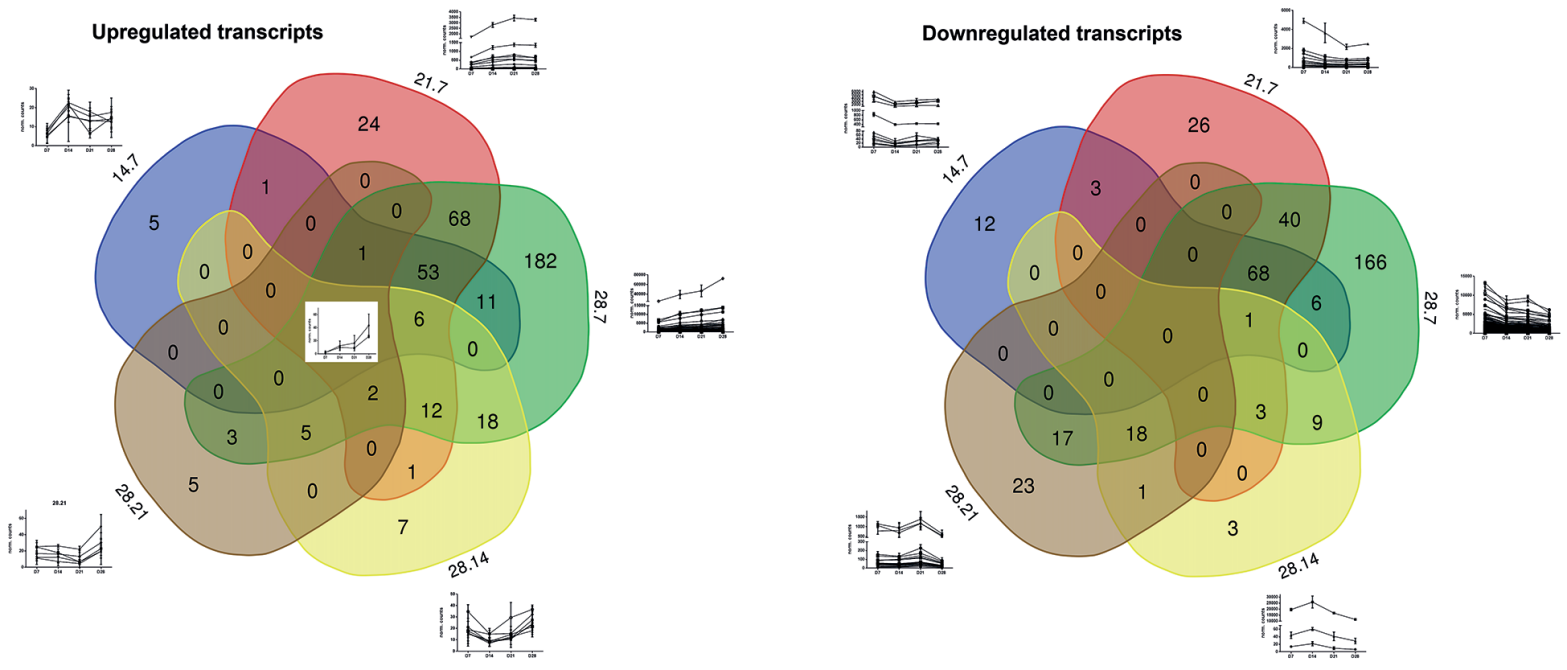
Venn diagrams showing the number of common differentially expressed genes (DEGs) between the different time points during prolonged starvation of Trypanosoma cruzi epimastigote culture. The time points selected during the prolonged starvation of *T. cruzi* epimastigote culture correspond to the exponential phase: day 7 (7); early stationary phase: day 14 (14); intermediate stationary phase: day 21 (21); and the final of the stationary phase: day 28 (28). The expression profile of DEGs belonging to the indicated compartments is shown in each graph as the mean of the normalised read count with its standard error at each time point from:^36^ 14.7 (Se vs E); 21.7 (Si vs E); 28.7 (Sf vs E); 28.14 (Sf vs Se); 28.21 (Sf vs Si).

**Fig. 3:**
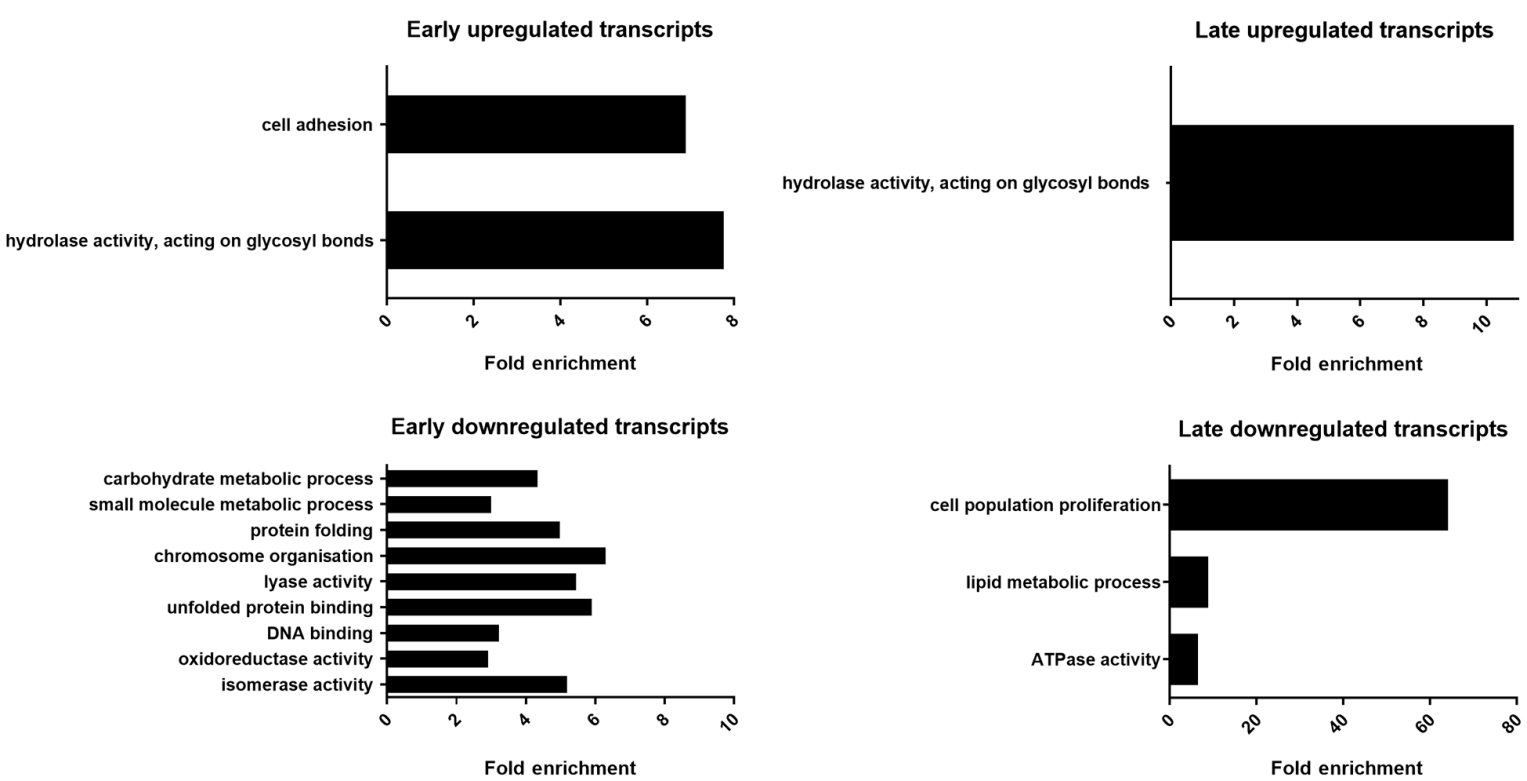
gene ontology (GO) analysis of early and late modulated transcripts (ERT and LRT respectively) identified during prolonged starvation of Trypanosoma cruzi epimastigote cultures. GO enrichment analysis (using GO Slim terms) was performed on TriTrypDB for early and late response DEGs up (upper panels) and down (bottom panels) regulated. The most significantly (p < 0.05) enriched GO terms in biological process and molecular function branches are presented.

The comparative analysis of DEGs between the selected time points: exponential phase (day 7, D7), early stationary phase (day 14, D14), intermediate stationary phase (day 21, D21), and final stationary phase (day 28, D28) is shown in Fig. 2. In order to distinguish temporal processes triggered by prolonged starvation within the long stationary phase of *T. cruzi* epimastigote culture, DEGs were classified as nutrient restriction early response transcripts (ERT) if significant different expression between the data derived from epimastigotes in the exponential (D7) and in the early stationary phase (D14) was observed (subclassified in Fig. 2 as following: D14 vs D7, D14 vs D7 plus D21 vs D7, D14 vs D7 and D21 vs D7 and D28 vs D7), likewise, DEGs were classified as late response transcripts (LRT) if the different expression was restricted to the changes between intermediate and late stationary phase (D28 vs D21) [Supplementary data (Fig. 2, Table I)]. GO analysis for ERT and LRT DEGs is shown in Fig. 3. Upregulated ERT show an enrichment of genes coding for proteins involved in cell adhesion and glycosyl bond hydrolase activity, while the downregulated ERT are enriched in genes coding for proteins involved in carbohydrate and small molecule metabolic process, protein folding, chromosome organisation, lyase activity, unfolded protein binding, oxidoreductase and isomerase activity and DNA binding. For LRT, we found an upregulation of genes for proteins with hydrolase activity of glycosyl bonds and a downregulation of genes coding for proteins involved in cell population proliferation, lipid metabolic processes, and ATPase activity. It is worth noting that the profiles of previously reported markers of the metacyclic trypomastigotes, genes coding for Metacyclin II (TcCLB.506529.600) and Metacyclin III (TcCLB.509251.6)^9^ ^39^ remain constant all along the prolonged starvation of the *T. cruzi* epimastigote culture not accompanying the slow increase of metacyclic forms described above [Supplementary data (Fig. 2)].

The GO terms enriched in the LRT are consistent with the quiescent status of the epimastigotes in the stationary phase and the decreased doubling time observed for the D28 parasite population [Supplementary data (Fig. 1)]. On the other hand, the GO terms enriched in the ERT can be interpreted as a rapid parasite adaptation triggered by incipient nutrient shortage that promotes the differentiation of parasites to infective stages. These findings prompt us to focus on the expression dynamics of protein-coding genes involved in adhesion, infectivity, and complement resistance.

Surface protein gene expression during prolonged starvation of *T. cruzi* epimastigote Surface proteins, arranged as large gene families exhibiting diversification and copy number diversity,^40^ play different roles in the parasite’s life cycle progression, in the host-cell interplay, immune system evasion and persistence of the parasite.^41^

**Fig. 4:**
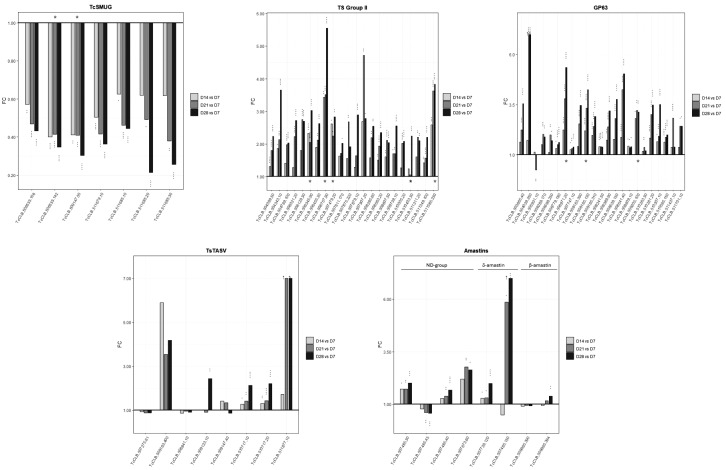
differential expression of transcripts coding for surface proteins identified during prolonged starvation of Trypanosoma cruzi epimastigote culture. The expression profile of genes of the indicated families of surface protein is shown. During the prolonged starvation of *T. cruzi* epimastigote culture time points at day 7, corresponding to the exponential phase, day 14, early stationary phase, day 21, intermediate stationary phase and day 28, the final of the stationary phase (D7, D14, D21 and D28 respectively) were selected for analysis. Light grey bars represent the expression at D14 relative to D7; grey bars the expression at D21 relative to D7 and black bars the expression at D28 relative to D7. Vertical asterisks over each bar indicate adjusted significance: * p < 0.05, ** p < 0.01, *** p < 0.001. Red and blue asterisks account for ERT and LRT, respectively. The arrow (↑) indicates the bar was truncated at FC = 7.

Among them, the surface proteins mucins and MASPs constitute a highly expressed gene family in *T. cruzi* ^42^ ^43^ involved in the parasite resistance against host immune system and attachment to the host cells.^44^ ^45^ Considering sequence similarities, the mucin family can be classified in TcMUC and TcSMUG.^44^ ^45^ While the TcMUC subfamily expression, as well as the MASPs’, are restricted to the parasite stages in the mammalian host,^46^ ^47^ the TcSMUG subfamily, is mainly expressed in the insect-dwelling stages.^48^ ^49^ The TcSMUG subfamily, in turn, is divided according to the size into two groups: small (S), composed of the 35-50 kDa mucins found in epimastigotes and metacyclic trypomastigotes, and large (L) which are not sialic acid acceptors and are only present in the surface of the epimastigote stage.^49^ All the annotated TcSMUG significantly diminish their expression, being TcSMUGL mostly ERT (2 out of 3) and maintain or exacerbate the profile along the long-lasting culture of *T. cruzi* [Fig. 4, Supplementary data (Table II)].

A different profile was found for the highly expressed trans-sialidase (TS) superfamily, one of the most expanded gene families in *T. cruzi*. ^50^ ^51^ ^52^ Recently, the TS catalytic activity proposed as a virulence factor has been confirmed and mutants lacking this activity cannot establish infection in mice.^53^ Although many TS superfamily proteins do not have TS catalytic activity, TS or TS-like genes were classified altogether into eight groups.^50^ ^51^ As for other superfamilies in *T. cruzi*, there is a high variability of the member numbers of TS among strains, from ~ 800 in CL Brener to ~2300 in Bug2148.^44^ ^45^ In CL Brener, TS group I includes ~19 catalytically active TS,^50^ ^51^ ^54^ namely TCNA and SAPA (shed acute-phase antigen) expressed in trypomastigotes, and TS-epi expressed in epimastigotes. Five genes from this group increase their expression relative to D7 [Supplementary data (Table II, Fig. 3)]. TS group II comprises a set of ~117 diverse GPI-anchored surface glycoproteins^50^ ^51^ expressed in the trypomastigote and intracellular amastigote forms of the parasites.^55^ ^56^ Proteins belonging to this group have been implicated in parasite adhesion and invasion of host cells.^56^ ^57^ ^58^ ^59^ Our transcriptomic analysis revealed that 46 transcripts for TS group II increased their expression relative to D7, (22 of them with significant values including 4 ERT) [Fig. 4, Supplementary data (Table II)]. Several genes coding for TS groups, including group III , which encompasses proteins involved in the complement system (see below), and group IV to VIII, with still unknown function,^50^ ^51^ also increase their expression in the prolonged nutrient restricted stationary parasite populations comparing with the exponential parasites [Supplementary data (Table II, Fig. 3)]. Besides, many ungrouped TS coding transcripts are also upregulated in the stationary growth phase [Supplementary data (Table I)].

GP63 metalloproteases also constitute a biologically relevant cell surface family of proteins involved in trypomastigote-host cell infection.^60^ ^61^ ^62^ Although the GP63 family is quite big, with more than 400 genes and pseudogenes, mRNAs corresponding to only 31 genes have been identified.^63^ Most show significantly upregulated expression, including 3 ERT [Fig. 4, Supplementary data (Table II)].

The expression of the TcTASV family, a group of surface proteins mainly expressed in bloodstream trypomastigotes,^64^ ^65^ also shows a gradual and significant increase in the expression of some members in the long stationary phase of the epimastigote growth culture during prolonged starvation [TcCLB.509123.10, TcCLB.510717.10, TcCLB.510717.20, TcCLB.511877.10, Fig. 4, Supplementary data (Table II)].

Amastins constitute another group of structurally related surface proteins first identified in *T. cruzi* ^66^ and then in Leishmania.^67^ Although several roles have been assigned to amastins, their exact role in infection and disease progression is still uncertain. These proteins have been reported to constitute one of the most immunogenic surface antigens^68^ ^69^ ^70^ ^71^ producing strong immune responses in humans^72^ ^73^ and therefore seem to be key proteins in the host-parasite interaction. Though four groups of amastins (α-, β-, γ- and δ) have been recognised, in *T. cruzi* only the existence of β- and δ-amastins has been reported.^74^ Nonetheless, the *T. cruzi* genome also bears annotated amastin genes, not assigned to β- or δ- amastin group. The expression of δ-amastins is restricted to amastigotes, whereas β-amastins are expressed in epimastigotes.^75^ We found that while the expression of the epimastigote β-amastin remains almost unchanged, the δ-amastin (TcCLB.507485.150) significantly increases the expression in the long stationary phase of the epimastigote growth culture during prolonged starvation [Fig. 4, Supplementary data (Table II)].

Although, it has been reported that using proteomic approaches for the recently differentiated epimastigotes derived from trypomastigotes,^35^ the upregulation of some surface proteins such as: a cruzipain protein group (TcCLB.507603.260, TcCLB.507603.270, TcCLB.509429.320 and TcCLB.509429.329), a GP63 (TcCLB.506435.370) and a trans-sialidase (TcCLB.509257.10), no significant upregulation was found for the encoding genes in the transcriptomic data here analysed.

Expression of genes involved in host complement resistance during prolonged starvation of *T. cruzi* epimastigote Considering the expression profile of surface proteins involved in the infectivity process during prolonged starvation of *T. cruzi* epimastigote, we wondered about the transcriptomic behaviour of genes coding for proteins involved in host complement bypass, one of the early mechanisms of innate immunity. The mechanisms governing complement resistance appear to be multifactorial, involving the expression of complement receptors on their surface. Parasites in their trypomastigote stage express several complement regulatory proteins^76^ and/or capture components with complement regulatory activity from the host bloodstream^77^ whose molecular inhibitory mechanisms are only partially understood.^78^ Among them, we focused on the calreticulin (TcCRT), the complement regulatory protein (TcCRP), and the trypomastigote decay-accelerating factor (T-DAF).

**Fig. 5:**
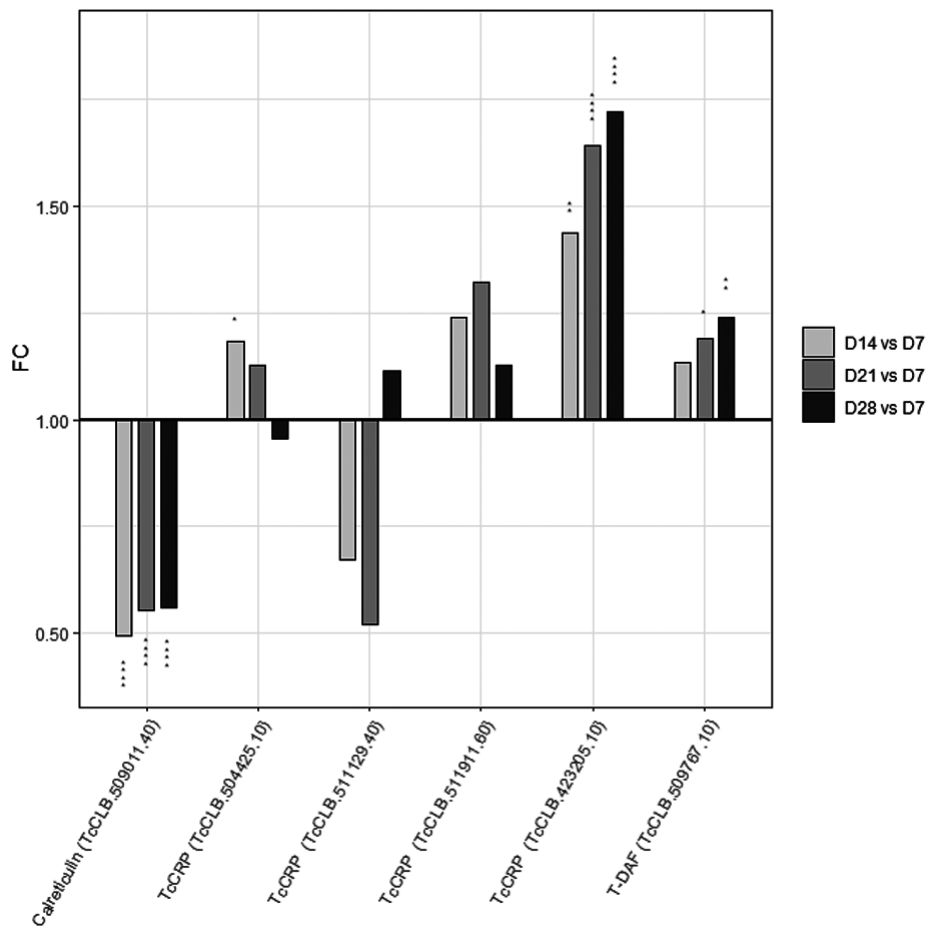
expression of genes involved in complement resistance during prolonged starvation of Trypanosoma cruzi epimastigote culture. The expression profile of genes of the indicated surface protein families is shown. During the prolonged starvation of *T. cruzi* epimastigote culture time points at day 7, corresponding to the exponential phase, day 14, early stationary phase, day 21, intermediate stationary phase and day 28, the final of the stationary phase (D7, D14, D21 and D28 respectively) were selected for analysis. Light grey bars represent the expression at D14 relative to D7; grey bars the expression at D21 relative to D7 and black bars the expression at D28 relative to D7. Vertical asterisks over each bar indicate adjusted significance: * p < 0.05, ** p < 0.01, *** p < 0.001.

TcCRT, originally named Tc45, is a multifunctional virulence factor that participates not only in the inhibition of classical and lectin complement system activation^79^ ^80^ ^81^ but also in the differentiation to the trypomastigote form.^82^ While transcriptomic analyses did not detect an increased expression of the TcCRT gene (TcCLB.509011.40) in the metacyclic trypomastigote compared to the epimastigote form,^15^ ^83^ higher expression was observed in the bloodstream trypomastigote,^83^ and in the intracellular amastigote form.^84^ Consistently with the reported metacyclic trypomastigote profile, we found an early drop of TcCRT gene expression that is maintained along the long stationary phase during prolonged starvation of *T. cruzi* epimastigote (Fig. 5).

TcCRP is a trypomastigote surface glycoprotein with sequence similarities to the TS family,^85^ that inhibits both classical and alternative complement system activation^86^ and shows a positive correlation between expression levels and strain virulence.^87^ The stable transfection of *T. cruzi* epimastigotes with TcCRP-10 cDNA confers complement resistance.^88^ TcCRP is encoded by the large FL-160 gene family^89^ and proteins encoded by these genes share sequence similarity with members of the TS group III.^50^ Members of this family (TcCLB.423205.10, TcCLB.504425.10, and TcCLB.511911.60) showed an increased transcript expression in the long stationary phase during prolonged starvation of *T. cruzi* epimastigote (Fig. 5).

Finally, T-DAF is an 87-93 kDa surface glycoprotein that inhibits both classical and alternative complement system activation (and probably also the lectin pathway)^90^ with higher expression (TcCLB.509767.10) in the metacyclic trypomastigote^15^ ^83^ and trypomastigote forms^84^ relative to the epimastigote forms. Here, a gradual increase in the expression of this gene in the D14, D21, and D28 parasite populations was found (Fig. 5).

**Fig. 6:**
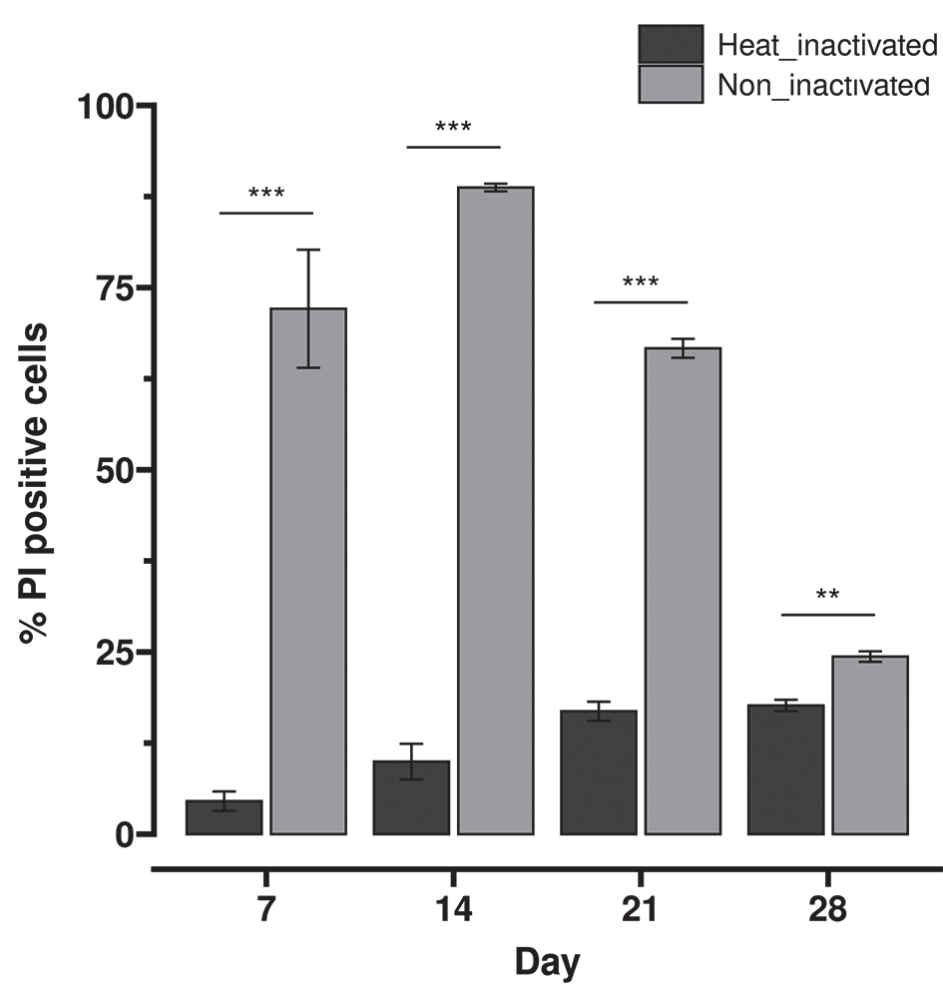
complement resistance during prolonged starvation of Trypanosoma cruzi epimastigote culture. Percentages of PI-positive parasites representing non-viable cells of the parasite populations from the prolonged starvation of *T. cruzi* epimastigote culture at day 7 (exponential phase), day 14 (early stationary phase), day 21 (intermediate stationary phase) and day 28 (final of the stationary phase) after incubation with human serum that had or had not been heat treated. At least three independent experiments were performed for each point analysed. Significant differences are indicated: ** = p < 0.01, *** = p <0.001.

The increased expression of genes encoding some complement evasion proteins suggests that the intermediate parasite forms during prolonged starvation of *T. cruzi* epimastigote may be able to bypass host complement growth inhibition. In order to understand the developmental pattern of *T. cruzi* complement resistance acquisition, we studied the cell viability of the D7, D14, D21 and D28 parasite populations after treatment with complement inactivated or non-inactivated human serum by flow cytometry using propidium iodide (PI) (Fig. 6). When the parasite populations were treated with heat-inactivated complement, a low percentage of PI-positive cells, indicative of cells with disrupted or absent membranes,^91^ was detected. In these conditions, maybe as a consequence of the nutrient restrictions, a slight increase in the percentage of PI labelled cells along the long stationary phase during prolonged starvation of *T. cruzi* epimastigote was observed. Conversely, after treatment with non-inactivated human serum, and in accordance with the reported complement susceptibility for *T. cruzi* epimastigotes,^92^ we found a high percentage of PI-labelled cells at D7. A similar pattern is observed at D14, supporting that complement resistance mechanisms are still mostly absent at the beginning of the long stationary phase during prolonged starvation of *T. cruzi* epimastigote. But later, in the intermediate stationary phase (D21), an increase in viable parasites is observed, leading to a parasite population mostly resistant to human complement at the final stationary phase (D28).

The increasing resistance to the host complement system of the parasite population in the long stationary phase during prolonged starvation of *T. cruzi* epimastigote culture suggests that the molecular mechanisms to evade the complement system acquired by the intermediate parasite forms are functioning.

Autophagy genes during prolonged starvation of *T. cruzi* epimastigote Since nutritional stress conditions constitute a strong stimulus for autophagy and differentiation in *T. cruzi*, being activated during,^4^ ^93^ ^94^ we studied the transcriptomic behaviour of genes coding for proteins involved in this process during prolonged starvation of *T. cruzi* epimastigote. Autophagy is a constitutive catabolic process responsible for self-degradation and reutilisation of the cell components, which is necessary to provide amino acids as the energy source for cell survival and to maintain cellular homeostasis, where portions of the cytoplasm are assembled in vesicles called autophagosomes that are fused with lysosomes.^92^ ^95^

**Fig. 7:**
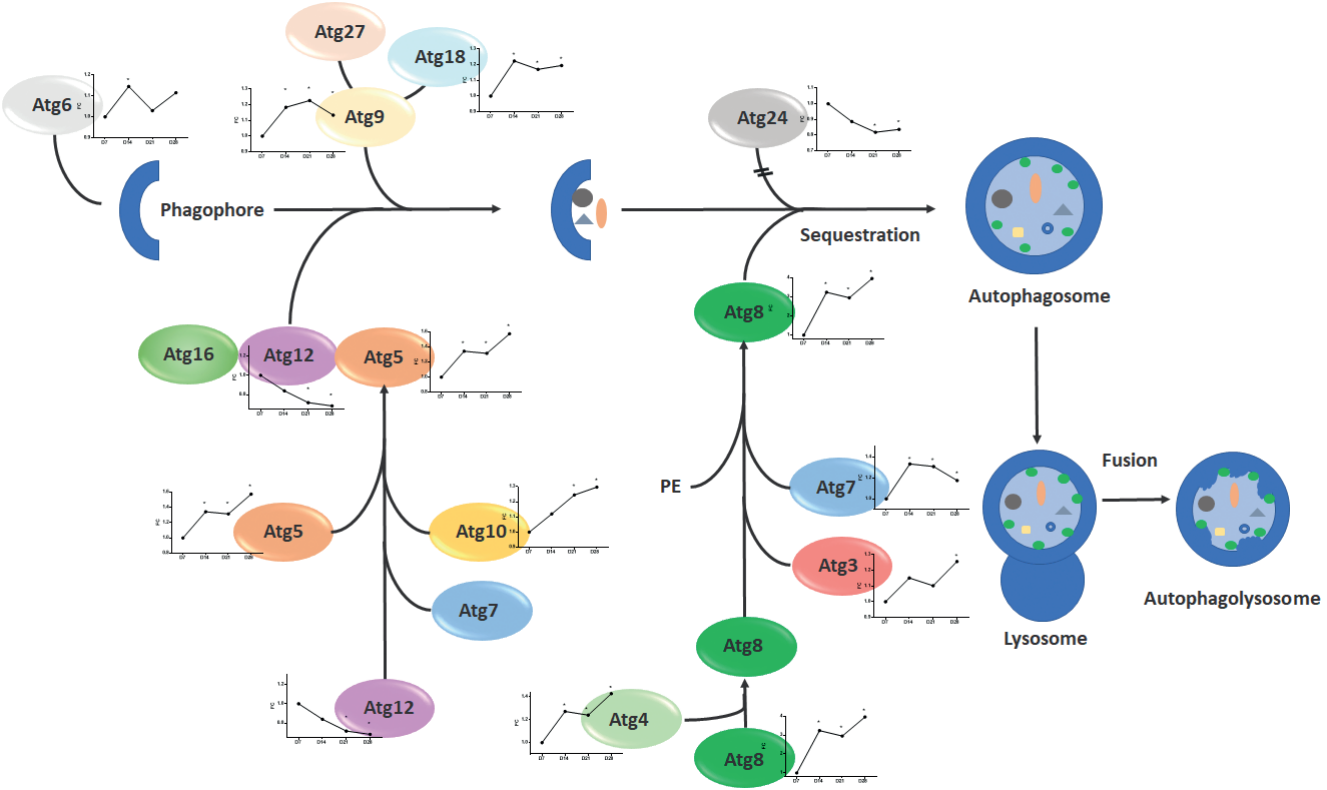
expression of genes involved in autophagy identified during prolonged starvation of Tryapnosma cruzi epimastigote culture. Autophagy-related genes (Atg) can be grouped according to their functions at key stages of the autophagy pathway. Atg6 is involved in the initiation of the phagophore. The phagophore elongation requires the sequential activation of Atg5-Atg12-Atg16 complex). During starvation, Atg8 is cleaved by Atg4 and conjugated with phosphatidylethanolamine (PE), to then be inserted in the autophagosome membrane during its maturation, contributing to vesicle elongation. Finally, the autophagosome fusion occurs with the lysosomes. The mean of the normalised read count with its standard error is shown in each graph along the prolonged starvation of *T. cruzi* epimastigote culture at day 7, corresponding to the exponential phase, day 14, early stationary phase, day 21, intermediate stationary phase and day 28, the final of the stationary phase (D7, D14, D21 and D28 respectively). Atg6 (TcCLB.507809.119), Atg27 (TcCLB.511529.59), Atg9 (TCCLB.506925.450), Atg18 (TcCLB.509669.100), Atg12 (TcCLB.511211.104), Atg7 (TcCLB.507711.150), Atg5 (TcCLB.509965.280), Atg10 (TcCLB.507389.50), Atg24 (TCCLB.510749.30), Atg8 (TcCLB.510533.180), Atg4 (TcCLB.509443.30 and TCCLB.511527.50), Atg3 (TcCLB.510257.90), Atg7 (TCCLB.507711.150).

The ubiquitin-like protein Atg8 that acts on vesicle expansion and completion,^63^ has two homologs in *T. cruzi*: TcAtg8.1 and TcAtg8.2.^94^ We found that expression of TcAtg8.2 gene (TcCLB.510533.180) increases during the long stationary phase of prolonged starvation of *T. cruzi* epimastigote culture. Similarly, mRNAs codifying for genes involved in the Atg8 conjugation, such as Atg7 (TcCLB.507711.150) and the Atg8 processing protein Atg4 (TcCLB.511527.50), also increase their expression. A summary of the expression profile of genes related to autophagy during the prolonged starvation of *T. cruzi* epimastigote culture is shown in Fig. 7 and Supplementary data (Fig. 4).

In conclusion, to further characterise the molecular changes accompanying the nutrient restriction within the insect dwelling parasite stage, we here deep on the analysis of the reported transcriptomic database derived from the parasite population obtained along the axenic growth of *T. cruzi* epimastigotes for more than 30 days without nutrient supplementation.^36^

In the assayed conditions, we observed the known co-existence of epimastigotes, metacyclic trypomastigotes as well as an increasing proportion of intermediate parasite forms with the nucleus-kinetoplast location characteristic of epimastigote and different growth resume ability.

To analyse the transcriptomic dynamics during prolonged starvation of *T. cruzi* epimastigote culture, we here discriminate early and late response transcripts. In addition, we delved into groups of genes not addressed in our previous work, which are of great importance in the parasite life cycle. We observed a rapid change in surface protein genes such as TS and GP63. Also, the expression of the surface proteins TcTASV and δ-amastin showed upregulation. In addition, an increasing expression of genes coding for autophagy (Atg4, Atg7, Atg8.2) and complement resistance (TcCRP and T-DAF) was found, and the latter was experimentally verified.

These results complement the analysis of processes that characterises the distinctive transcriptomic we reported for transitional parasites obtained along the axenic growth of *T. cruzi* epimastigotes for more than 30 days without nutrient supplementation,^36^ supporting previous proposals of the existence of a specific parasite stage that morphologically resembles epimastigotes but exhibits distinctive biological characteristics.

## SUPPLEMENTARY MATERIALS

Supplementary material

## Data Availability

A publicly available dataset was analysed in this study. This dataset can be found in the National Centre for Biotechnology (NCBI), Sequence Read Archive (SRA) BioProject ID PRJNA915394.
