# Supplementary material for: Transcriptomic dynamics reveals sequential acquisition of complement resistance during prolonged starvation of Trypanosoma cruzi epimastigote
Source: Mem Inst Oswaldo Cruz. 2026 Mar 6;121:e250127. doi: 10.1590/0074-02760250127 (PMC12965721; doi:10.1590/0074-02760250127)
Supplement: Supplementary material [file 1678-8060-mioc-121-e250127-s1.pdf]

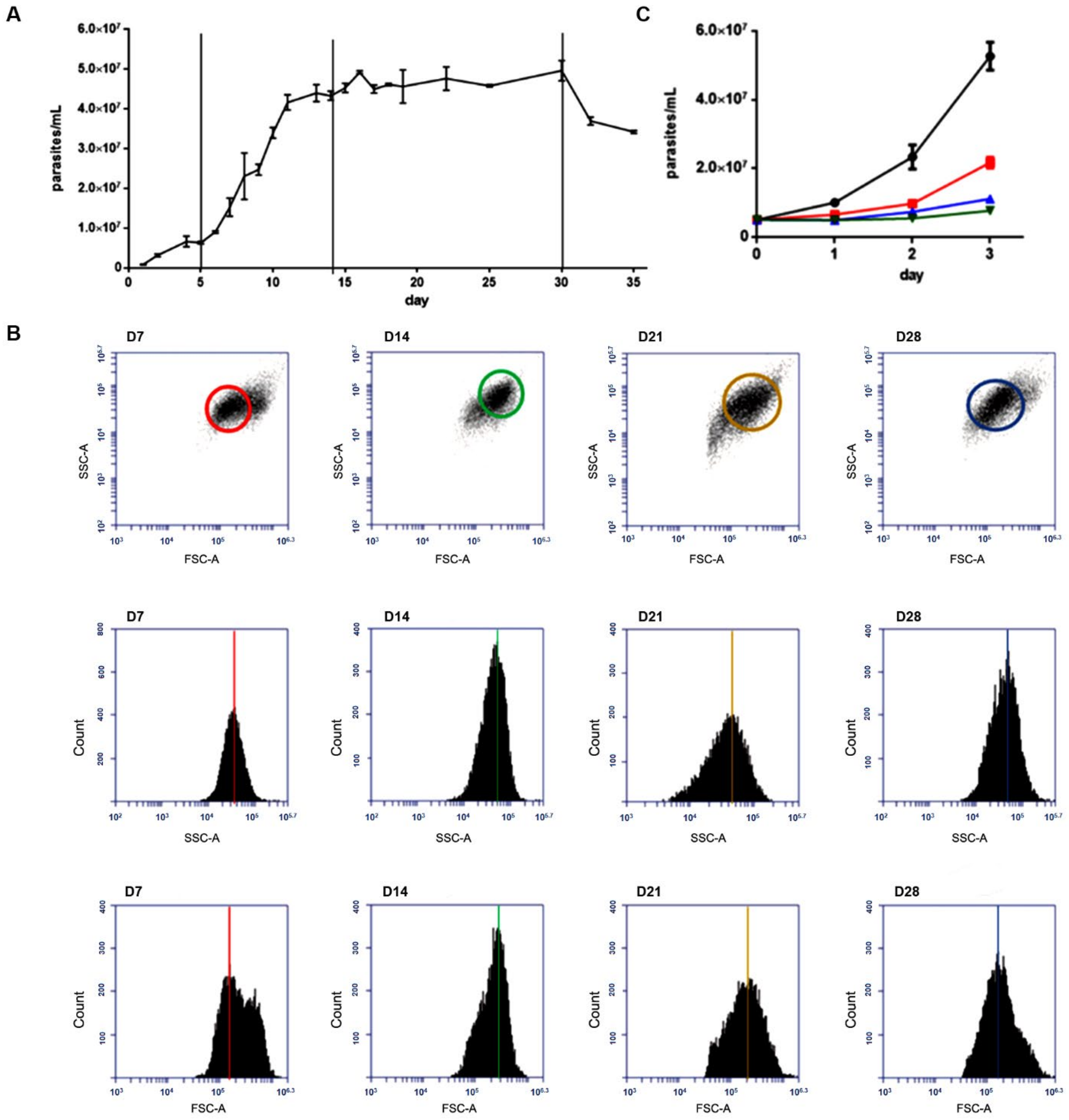

Fig. 1: morphology and growth kinetics during prolonged starvation of *Trypanosoma cruzi* epimastigote culture. (A) *In vitro* growth curve of *T. cruzi* epimastigote in BHI medium supplemented with 10% SBF at 28°C. Vertical bars delimit different growth stages: lag, exponential, stationary, and death phases. (B) Morphology analysis of parasite populations from day 7 (D7), day 14 (D14), day 21 (D21) and day 28 (D28) were selected for analysis using BD Accuri C6 flow cytometry. Upper panel: Forward scatter (FSC) versus side scatter (SSC) dot plots; middle panel: SSC analysis; bottom panel: FSC analysis. (C) Growth curve of parasites from selected points (D7: circles; D14: squares; D21: triangles; D28: inverted triangles) after dilution to a concentration of 1 x 10^6 cells/mL in fresh BHI medium. Results of direct cell count of at least three independent cultures ± standard deviation (SD) are shown.

CC BY

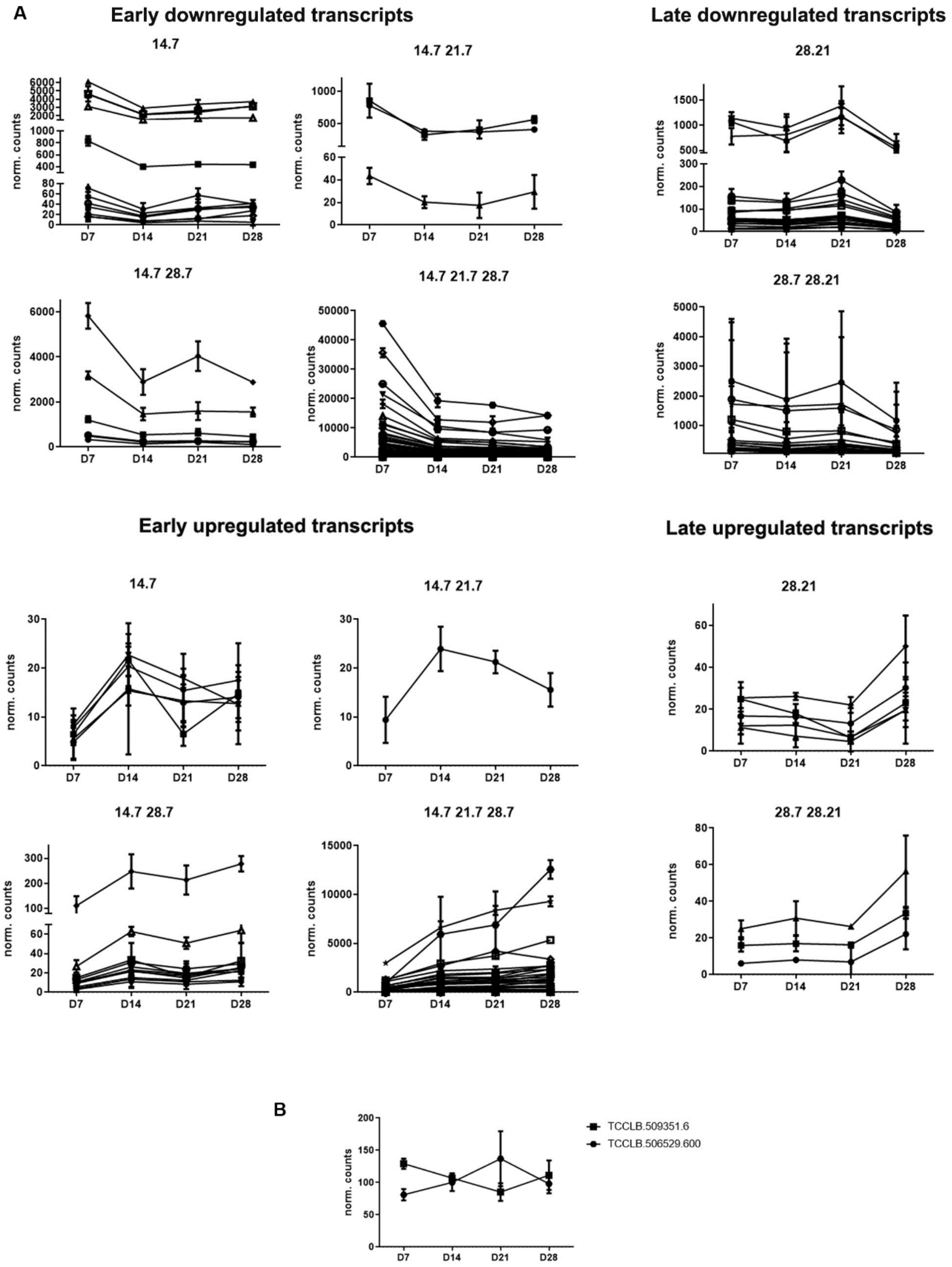

Fig. 2: gene expression profile of early and late modulated transcripts (ERT and LRT respectively) identified during prolonged starvation of *Trypanosoma cruzi* epimastigote culture. The mean of the normalised read count with its standard error is shown in each graph at day 7(D7), exponential phase; day 14 (D14), early stationary phase; day 21 (D21), intermediate stationary phase; and day 28 (D28), final stationary phase. (A) ERT includes 14.7 (DEGs between D14 vs D7); 14.7 21.7 (DEGs between D14 vs D7 and D21 vs D7); and 14.7 21.7 28.7 (DEGs between D14 vs D7, D21 vs D7 and D28 vs D7). LRT includes 28.21 (DEGs between D21 and D28). (B) Expression profile of transcripts for Metacyclin II (TcCLB.506529.600) and Metacyclin III (TcCLB.509251.6).

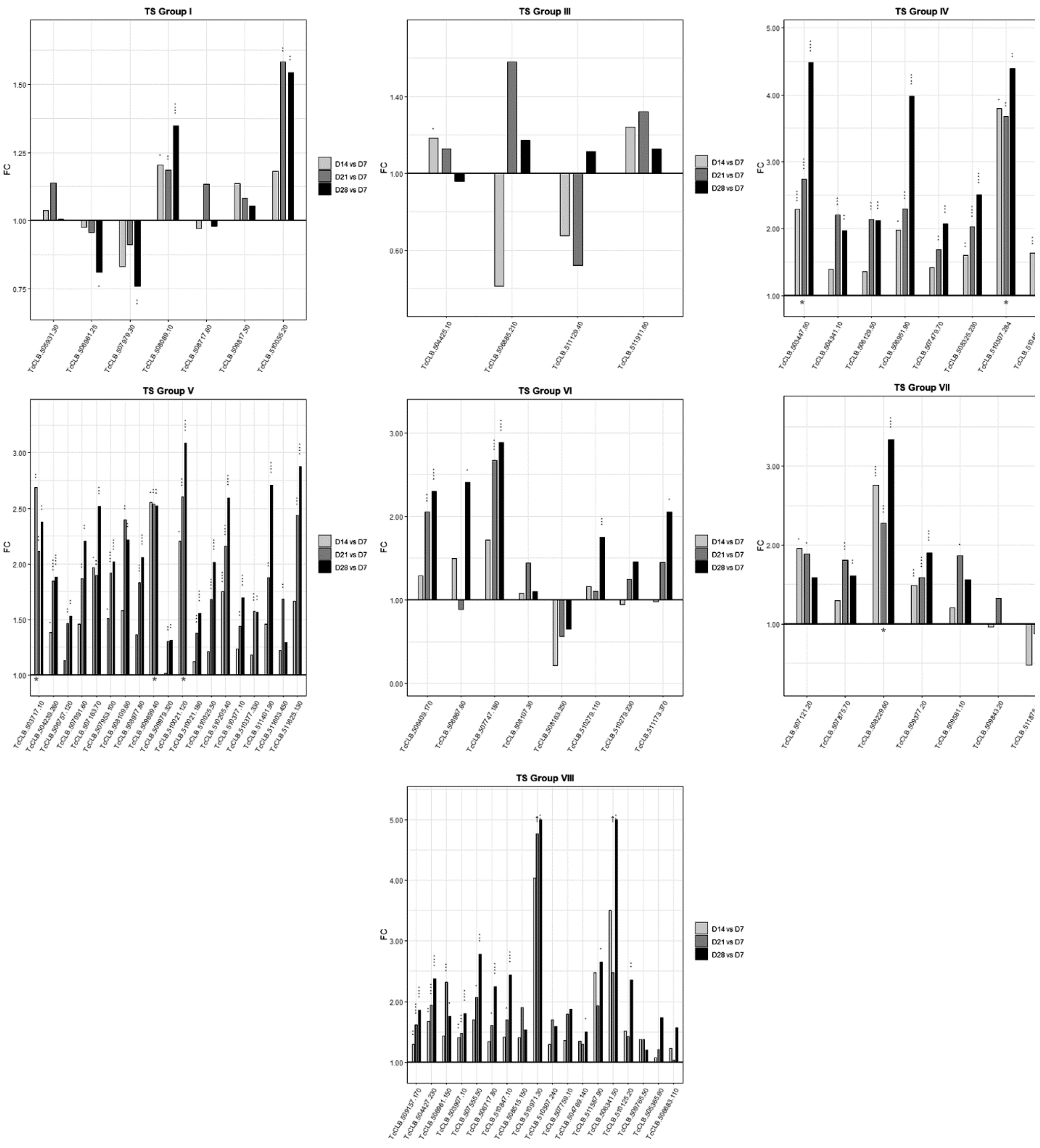

Fig. 3: differential expression of transcripts coding for trans-sialidase (TS) superfamily identified during prolonged starvation of *Trypanosoma cruzi* epimastigote culture. The expression profile of genes of the indicated surface protein families is shown. During the prolonged starvation of *T. cruzi* epimastigote culture time points at day 7, corresponding to the exponential phase, day 14, early stationary phase, day 21, intermediate stationary phase and day 28, the final of the stationary phase (D7, D14, D21 and D28 respectively) were selected for analysis. Light grey bars represent the expression at D14 relative to D7; grey bars the expression at D21 relative to D7 and black bars the expression at D28 relative to D7. Vertical asterisks over each bar indicate adjusted significance: * $p < 0.05$, ** $p < 0.01$, *** $p < 0.001$. Red asterisks account for early modulated transcripts ERT. The arrow (↑) indicates the bar was truncated at FC = 5.

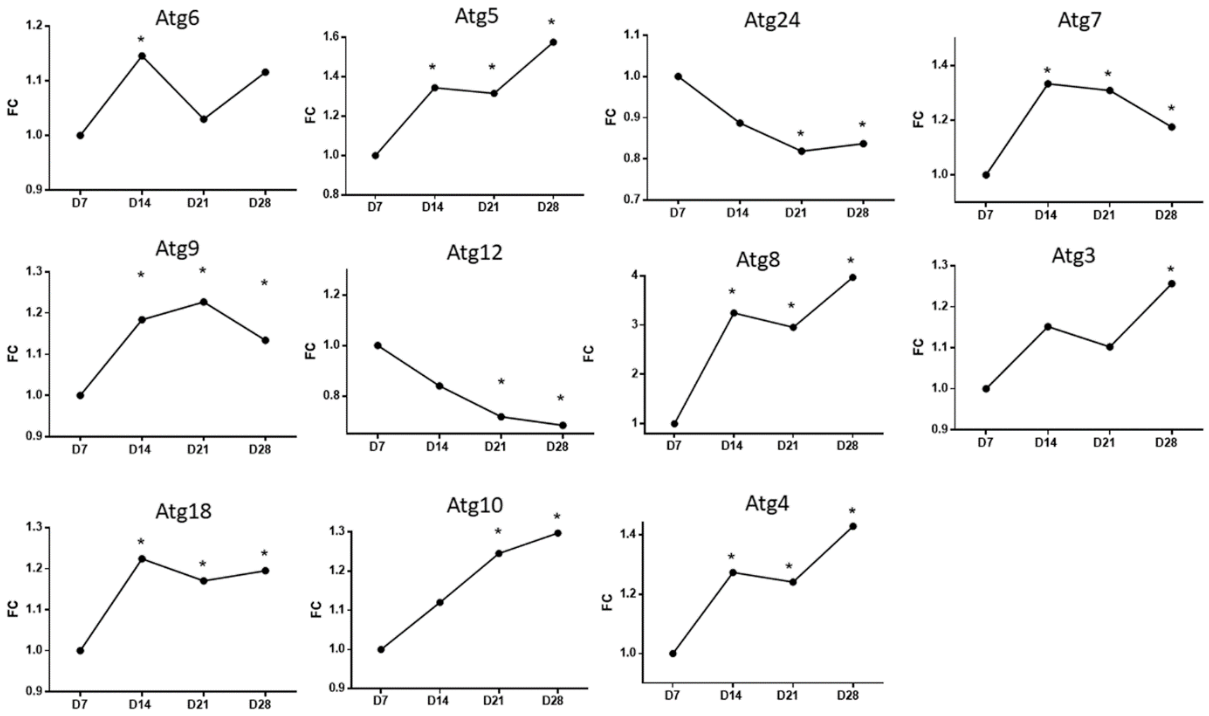

Fig. 4: fold change (FC) of transcripts from autophagy-related genes across prolonged starvation of *Trypanosoma cruzi* epimastigote culture. FC values for each gene transcript in the transcriptome datasets at day 7, corresponding to the exponential phase, day 14, early stationary phase, day 21, intermediate stationary phase and day 28, the final of the stationary phase (D7, D14, D21 and D28 respectively) are shown. Statistically significant variations in FC relative to day 7 are indicated with an asterisk (*).

TABLE I

Up and downregulated nutrient restriction response transcripts along the axenic growth of *Trypanosoma cruzi* epimastigotes for more than 30 days without nutrient supplementation

UPREGULATED			
Day comparison	n	gene ID	Gene name
14.7 21.7 28.14 28.21 28.7	2	TcCLB.506525.120	NLI interacting factor-like phosphatase, putative
		TcCLB.503885.100	hypothetical protein, conserved
14.7 21.7 28.14 28.7	6	TcCLB.506971.20	surface protease GP63, putative
		TcCLB.507907.10	trans-sialidase, putative
		TcCLB.503865.12	small nuclear RNA (snRNA) U6
		TcCLB.503745.30	ascorbate peroxidase
		TcCLB.511693.130	hypothetical protein
		TcCLB.507953.60	hypothetical protein
14.7 21.7 28.21 28.7	1	TcCLB.507995.20	trans-sialidase, putative
21.7 28.14 28.21 28.7	2	TcCLB.510409.10	STE/STE11 serine/threonine-protein kinase, putative
		TcCLB.511191.39	small nuclear RNA (snRNA) U2
14.7 21.7 28.7	53	TcCLB.508989.60	chaperone protein DnaJ, putative
		TcCLB.510601.10	hypothetical protein, conserved
		TcCLB.511621.110	hypothetical protein
		TcCLB.509699.40	trans-sialidase, Group V, putative
		TcCLB.511585.230	trans-sialidase, Group II, putative
		TcCLB.507677.160	hypothetical protein, conserved
		TcCLB.506967.65	mucin TcMUCII (pseudogene), putative
		TcCLB.508923.10	amino acid permease, putative
		TcCLB.509967.20	mismatch repair protein MSH4, putative
		TcCLB.510441.41	trans-sialidase (pseudogene), putative
		TcCLB.506203.10	nucleoside transporter 1, putative
		TcCLB.506495.50	hypothetical protein
		TcCLB.507807.30	hypothetical protein
		TcCLB.506859.230	hypothetical protein, conserved
		TcCLB.511325.40	amino acid permease, putative
		TcCLB.508229.60	trans-sialidase, Group VII, putative

		TcCLB.506925.10	hypothetical protein
		TcCLB.509707.10	protein associated with differentiation 8, putative
		TcCLB.510021.120	trans-sialidase, Group V, putative
		TcCLB.506625.190	hypothetical protein, conserved
		TcCLB.503501.50	trans-sialidase (pseudogene), putative
		TcCLB.504117.50	hypothetical protein, conserved (pseudogene)
		TcCLB.506345.90	trans-sialidase, Group II, putative
		TcCLB.510013.59	trans-sialidase (pseudogene), putative
		TcCLB.507479.20	trans-sialidase, Group II, putative
		TcCLB.507811.100	amino acid permease, putative
		TcCLB.509205.100	surface protease GP63, putative
		TcCLB.504427.244	small nuclear RNA (snRNA) U3
		TcCLB.506671.10	Mucin-associated surface protein (MASP), subgroup S020
		TcCLB.511325.25	amino acid transporter, putative
		TcCLB.510741.70	STE/STE11 serine/threonine-protein kinase, putative
		TcCLB.510533.180	microtubule-associated protein 1A/1B, light chain 3, putative
		TcCLB.508165.130	surface protease GP63, putative
		TcCLB.510103.24	hypothetical protein, conserved
		TcCLB.503447.50	trans-sialidase, Group IV, putative
		TcCLB.504153.260	Flagellum attachment zone protein 12
		TcCLB.511325.50	amino acid permease, putative
		TcCLB.404975.30	trans-sialidase (pseudogene), putative
		TcCLB.503717.10	trans-sialidase, Group V, putative
		TcCLB.509203.14	hypothetical protein
		TcCLB.507467.10	receptor-type adenylate cyclase, putative (fragment)
		TcCLB.507465.10	receptor-type adenylate cyclase, putative (fragment)
		TcCLB.507707.20	hypothetical protein, conserved
		TcCLB.508831.66	hypothetical protein, conserved
		TcCLB.508127.5	hypothetical protein, conserved
		TcCLB.510307.284	trans-sialidase, Group IV, putative
		TcCLB.511127.50	hypothetical protein, conserved
		TcCLB.507143.90	hypothetical protein

		TcCLB.506757.90	trans-sialidase, Group II, putative
		TcCLB.507101.10	amino acid permease, putative
		TcCLB.511911.30	mismatch repair protein MSH4 (pseudogene), putative
		TcCLB.509449.10	receptor-type adenylate cyclase, putative
		TcCLB.503683.30	RNA-binding protein, putative
21.7 28.14 28.7	12	TcCLB.510015.10	hypothetical protein
		TcCLB.506951.90	trans-sialidase, Group IV, putative
		TcCLB.503665.40	surface protease GP63 (pseudogene), putative
		TcCLB.503683.11	Double RNA binding domain protein 6A
		TcCLB.511691.20	serine sulfhydrylase
		TcCLB.508541.40	surface protease GP63 (pseudogene), putative
		TcCLB.508539.150	surface protease GP63 (pseudogene), putative
		TcCLB.504039.250	surface protease GP63 (pseudogene), putative
		TcCLB.510105.65	small nuclear RNA (snRNA) U4
		TcCLB.508163.330	surface protease GP63, putative
		TcCLB.506021.20	trans-sialidase, Group II, putative
		TcCLB.506925.170	hypothetical protein, conserved
28.14 28.21 28.7	5	TcCLB.510297.10	hypothetical protein, conserved
		TcCLB.509595.20	hypothetical protein, conserved
		TcCLB.509123.10	Trypomastigote, Alanine, Serine and Valine rich protein (TASV), subfamily C
		TcCLB.506755.200	hypothetical protein, conserved
		TcCLB.447847.10	trans-sialidase (pseudogene), putative
14.7 21.7	1	TcCLB.509267.3	receptor-type adenylate cyclase, putative
14.7 28.7	11	TcCLB.507699.170	surface protease GP63 (pseudogene), putative
		TcCLB.507047.40	trans-sialidase, Group II, putative
		TcCLB.506289.210	surface protease GP63, putative
		TcCLB.510373.90	Mucin-associated surface protein (MASP), subgroup S079
		TcCLB.509705.10	cellulosomal scaffoldin anchoring protein, putative
		TcCLB.506967.150	trans-sialidase (pseudogene), putative
		TcCLB.509979.200	Mucin-associated surface protein (MASP), subgroup S008
		TcCLB.506289.200	hypothetical protein
		TcCLB.507957.320	Mucin-associated surface protein (MASP), subgroup S068

		TcCLB.510851.40	trans-sialidase (pseudogene), putative
		TcCLB.507143.60	hypothetical protein
21.7 28.7	68	TcCLB.508215.3	CMGC/SRPK protein kinase, putative
		TcCLB.510407.9	Serine/threonine-protein kinase NEK11, putative
		TcCLB.509767.40	pre-mRNA splicing factor, putative
		TcCLB.507711.110	Surface membrane protein
		TcCLB.509205.110	elongation factor G2-like protein, putative
		TcCLB.508389.90	surface protease GP63, putative
		TcCLB.511173.190	trans-sialidase (pseudogene), putative
		TcCLB.507699.230	trans-sialidase, putative
		TcCLB.510275.370	Mucin-associated surface protein (MASP), subgroup S136
		TcCLB.504051.20	ATP-dependent DNA DEAD/DEAH box helicase, putative
		TcCLB.506409.170	trans-sialidase, Group VI, putative
		TcCLB.509265.120	trans-sialidase, putative
		TcCLB.510533.90	hypothetical protein, conserved
		TcCLB.507957.90	mucin TcMUCII, putative
		TcCLB.506861.10	ATP-dependent DEAD/H RNA helicase, putative
		TcCLB.507275.20	hypothetical protein, conserved
		TcCLB.510309.70	hypothetical protein, conserved
		TcCLB.510491.60	trans-sialidase, Group IV, putative
		TcCLB.477079.10	protein kinase, putative
		TcCLB.509805.50	AN1-like zinc finger, putative
		TcCLB.510127.70	leucine-rich repeat protein 1 (LRRP1), putative
		TcCLB.507555.50	trans-sialidase, Group VIII, putative
		TcCLB.504343.10	trans-sialidase, Group II, putative
		TcCLB.505807.130	Glutamine amidotransferase class-I, putative
		TcCLB.508109.60	trans-sialidase, Group V, putative
		TcCLB.508325.230	trans-sialidase, Group IV, putative
		TcCLB.504087.10	Ring finger domain containing protein, putative
		TcCLB.510877.140	hypothetical protein, conserved
		TcCLB.510205.40	trans-sialidase, Group V, putative
		TcCLB.511625.130	trans-sialidase, Group V, putative

		TcCLB.506183.90	trans-sialidase (pseudogene), putative
		TcCLB.503841.70	aspartate aminotransferase, putative
		TcCLB.511303.40	carnitine O-acetyltransferase, putative
		TcCLB.506455.30	trans-sialidase, Group II, putative
		TcCLB.511431.60	hypothetical protein, conserved
		TcCLB.511311.20	trans-sialidase, Group II, putative
		TcCLB.418405.30	trans-sialidase, putative
		TcCLB.507673.60	Amastin surface glycoprotein, putative
		TcCLB.510023.10	hypothetical protein, conserved (pseudogene)
		TcCLB.506677.4	hypothetical protein, conserved
		TcCLB.506775.30	tubulin-tyrosine ligase-like protein, putative
		TcCLB.507079.20	dynein heavy chain, cytosolic, putative
		TcCLB.511711.40	hypothetical protein, conserved
		TcCLB.511127.80	hypothetical protein, conserved
		TcCLB.510565.70	STE/STE11 serine/threonine-protein kinase, putative
		TcCLB.506129.30	trans-sialidase, Group II, putative
		TcCLB.506795.10	RNA-binding protein, putative
		TcCLB.506779.188	H/ACA snoRNA, TC6C1H3
		TcCLB.510281.20	surface protease GP63, putative
		TcCLB.506129.50	trans-sialidase, Group IV, putative
		TcCLB.507485.150	amastin, putative
		TcCLB.511593.91	trans-sialidase, putative (fragment)
		TcCLB.510241.20	hypothetical protein, conserved
		TcCLB.404711.10	trans-sialidase (pseudogene), putative
		TcCLB.508607.50	trans-sialidase, Group II, putative
		TcCLB.510005.20	trans-sialidase, Group II, putative
		TcCLB.504219.40	trans-sialidase (pseudogene), putative
		TcCLB.508323.90	hypothetical protein, conserved
		TcCLB.507747.180	trans-sialidase, Group VI, putative
		TcCLB.511445.120	hypothetical protein, conserved
		TcCLB.507617.100	C/D small nucleolar RNA (snoRNA), TB9C2C6
		TcCLB.506401.290	hypothetical protein, conserved

		TcCLB.508285.60	trans-sialidase, Group II, putative
		TcCLB.503715.40	STE/STE11 serine/threonine-protein kinase, putative
		TcCLB.510323.110	NLI interacting factor-like phosphatase, putative
		TcCLB.508165.310	surface protease GP63, putative
		TcCLB.507089.30	Zinc finger protein, C3H1 type-like
		TcCLB.510243.80	hypothetical protein, conserved
21.7 28.14	1	TcCLB.506765.49	hypothetical protein
28.14 28.7	18	TcCLB.511603.200	mucin TcMUCII, putative
		TcCLB.506717.130	trans-sialidase (pseudogene), putative
		TcCLB.507879.10	trans-sialidase, Group II, putative
		TcCLB.506285.40	Mucin-associated surface protein (MASP) (pseudogene)
		TcCLB.507511.50	hypothetical protein
		TcCLB.510275.160	trans-sialidase (pseudogene), putative
		TcCLB.506737.90	trans-sialidase, Group V, putative
		TcCLB.511607.60	trans-sialidase (pseudogene), putative
		TcCLB.510739.30	C/D small nucleolar RNA (snoRNA), TB9C3C2
		TcCLB.510281.10	hypothetical protein
		TcCLB.506357.21	H/ACA snoRNA, TC10C4H2
		TcCLB.506285.30	trans-sialidase (pseudogene), putative
		TcCLB.509007.40	hypothetical protein, conserved
		TcCLB.510427.10	hypothetical protein, conserved
		TcCLB.510295.59	hypothetical protein, conserved
		TcCLB.510307.10	surface protease GP63, putative
		TcCLB.447925.10	cystathionine beta-synthase, putative (fragment)
		TcCLB.507639.30	universal minicircle sequence binding protein 1
28.21 28.7	3	TcCLB.510403.30	trans-sialidase, Group II, putative
		TcCLB.510049.10	trans-sialidase, putative
		TcCLB.509699.110	Mucin-associated surface protein (MASP), subgroup S002
14.7	5	TcCLB.511127.10	RNA-binding protein 5, putative
		TcCLB.400945.10	trans-sialidase (pseudogene), putative
		TcCLB.511413.19	trans-sialidase, putative
		TcCLB.509977.15	surface protease GP63 (pseudogene), putative

		TcCLB.507485.159	tuzin (fragment)
21.7	24	TcCLB.506847.50	trans-sialidase (pseudogene), putative
		TcCLB.506529.595	hypothetical protein
		TcCLB.506961.150	trans-sialidase, Group VIII, putative
		TcCLB.506767.40	Mucin-associated surface protein (MASP), subgroup S104
		TcCLB.506529.480	Fusaric acid resistance protein-like, putative
		TcCLB.504149.210	N-acetyltransferase complex ARD1 subunit (pseudogene), putative
		TcCLB.507527.50	cation transporter, putative
		TcCLB.508323.199	ATP-dependent DEAD/H RNA helicase, putative (fragment)
		TcCLB.507907.20	trans-sialidase, Group II, putative
		TcCLB.506341.40	hypothetical protein
		TcCLB.506767.209	syntaxin binding protein (pseudogene), putative
		TcCLB.508089.20	UDP-glucoronosyl and UDP-glucosyl transferase, putative
		TcCLB.506495.30	retrotransposon hot spot protein (RHS, pseudogene), putative
		TcCLB.507875.220	trans-sialidase, Group II, putative
		TcCLB.504341.10	trans-sialidase, Group IV, putative
		TcCLB.506205.10	hypothetical protein, conserved
		TcCLB.504427.20	hypothetical protein, conserved
		TcCLB.506289.170	surface protease GP63, putative
		TcCLB.504035.149	kinesin, putative
		TcCLB.507237.220	Mucin-associated surface protein (MASP), subgroup S053
		TcCLB.511709.19	phosphatidylinositol 3-kinase catalytic subunit, putative (fragment)
		TcCLB.511569.30	trans-sialidase (pseudogene), putative
		TcCLB.507881.110	trans-sialidase (pseudogene), putative
		TcCLB.511437.10	surface protease GP63, putative
28.7	182	TcCLB.507747.130	trans-sialidase (pseudogene), putative
		TcCLB.507981.10	trans-sialidase (pseudogene), putative
		TcCLB.507923.20	U3 small nuclear ribonucleoprotein (snRNP), putative
		TcCLB.509341.20	hypothetical protein, conserved
		TcCLB.506147.20	hypothetical protein
		TcCLB.504643.30	ubiquitin-conjugating enzyme E2, putative
		TcCLB.509819.20	hypothetical protein, conserved

		TcCLB.506505.30	hypothetical protein, conserved
		TcCLB.508307.134	hypothetical protein, conserved
		TcCLB.509581.50	hypothetical protein
		TcCLB.506661.10	fatty acid elongase, putative
		TcCLB.511599.10	trans-sialidase, putative
		TcCLB.506369.40	hypothetical protein, conserved
		TcCLB.510717.10	Trypomastigote, Alanine, Serine and Valine rich protein (TASV), subfamily A (fragment)
		TcCLB.507835.10	trans-sialidase (pseudogene), putative
		TcCLB.506625.230	hypothetical protein, conserved
		TcCLB.510971.30	trans-sialidase, Group VIII, putative
		TcCLB.509765.129	trans-sialidase, putative (fragment)
		TcCLB.507959.180	surface protease GP63 (pseudogene), putative
		TcCLB.504769.100	trans-sialidase, Group II, putative
		TcCLB.506543.113	hypothetical protein, conserved
		TcCLB.506481.30	hypothetical protein, conserved
		TcCLB.510847.10	trans-sialidase, Group VIII, putative
		TcCLB.510369.10	Mucin-associated surface protein (MASP), subgroup S061
		TcCLB.511349.110	retrotransposon hot spot protein (RHS, pseudogene), putative
		TcCLB.507389.10	Flagellum attachment zone protein 14
		TcCLB.510125.20	trans-sialidase, Group VIII, putative
		TcCLB.509441.10	hypothetical protein, conserved
		TcCLB.511431.90	conserved protein, unknown function
		TcCLB.511369.10	elongation factor 1-alpha, putative
		TcCLB.511349.100	trans-sialidase, Group II, putative
		TcCLB.509707.20	hypothetical protein, conserved
		TcCLB.511411.16	Spinocerebellar ataxia type 10 protein domain containing protein, putative
		TcCLB.509669.170	Nodulin-like, putative
		TcCLB.509739.10	trans-sialidase, Group II, putative
		TcCLB.506411.25	hypothetical protein, conserved (pseudogene)
		TcCLB.506399.60	hypothetical protein, conserved
		TcCLB.511567.20	retrotransposon hot spot protein (RHS, pseudogene), putative
		TcCLB.510717.20	Trypomastigote, Alanine, Serine and Valine rich protein (TASV), subfamily A

		TcCLB.506375.35	hypothetical protein
		TcCLB.507957.260	surface protease GP63 (pseudogene), putative
		TcCLB.421173.4	trans-sialidase, putative (fragment)
		TcCLB.509167.140	RNA-binding protein 42 (RNA-binding motif protein 42), putative
		TcCLB.507611.170	trans-sialidase, Group II, putative
		TcCLB.510961.30	Mucin-associated surface protein (MASP), subgroup S013
		TcCLB.508343.10	trans-sialidase, putative
		TcCLB.508775.20	retrotransposon hot spot protein (RHS, pseudogene), putative
		TcCLB.504029.101	hypothetical protein, conserved
		TcCLB.507121.11	trans-sialidase, putative (fragment)
		TcCLB.507091.160	trans-sialidase (pseudogene), putative
		TcCLB.510025.50	trans-sialidase, Group V, putative
		TcCLB.504229.110	amino acid permease 24, putative
		TcCLB.507091.60	trans-sialidase, Group V, putative
		TcCLB.507617.160	C/D small nucleolar RNA (snoRNA), TB9C2C3
		TcCLB.503613.10	Calcium/calmodulin-dependent protein kinase kinase, putative
		TcCLB.508687.30	Mucin-associated surface protein (MASP) (pseudogene)
		TcCLB.506599.330	Mucin-associated surface protein (MASP), subgroup S001
		TcCLB.507873.30	Double RNA binding domain protein 7
		TcCLB.438923.10	hypothetical protein (fragment)
		TcCLB.510241.110	Pescadillo N-terminus/BRCA1 C Terminus (BRCT) domain containing protein, putative
		TcCLB.509197.10	cation transporter, putative
		TcCLB.506407.10	lysosomal alpha-mannosidase precursor, putative
		TcCLB.511173.470	trans-sialidase, Group V, putative
		TcCLB.504055.130	trans-sialidase (pseudogene), putative
		TcCLB.511433.10	hypothetical protein, conserved
		TcCLB.506623.20	trans-sialidase (pseudogene), putative
		TcCLB.508277.350	cation transporter, putative
		TcCLB.504099.50	trans-sialidase, Group II, putative
		TcCLB.507099.60	hypothetical protein, conserved
		TcCLB.503441.5	trans-sialidase, putative (fragment)
		TcCLB.506973.140	hypothetical protein

		TcCLB.506885.354	hypothetical protein, conserved
		TcCLB.508121.30	trans-sialidase (pseudogene), putative
		TcCLB.507485.120	tuzin
		TcCLB.507163.70	trans-sialidase, Group V, putative
		TcCLB.507957.220	Mucin-associated surface protein (MASP), subgroup S078
		TcCLB.506633.90	folate/pteridine transporter, putative
		TcCLB.401569.10	trans-sialidase, putative
		TcCLB.504239.434	trans-sialidase, putative (fragment)
		TcCLB.504213.100	hypothetical protein, conserved
		TcCLB.503957.10	trans-sialidase, putative
		TcCLB.505155.4	trans-sialidase, putative (fragment)
		TcCLB.509099.40	hypothetical protein, conserved
		TcCLB.510205.10	trans-sialidase, putative
		TcCLB.511401.90	trans-sialidase, Group V, putative
		TcCLB.506859.204	hypothetical protein, conserved
		TcCLB.503659.30	trans-sialidase, putative
		TcCLB.506499.10	hypothetical protein, conserved (pseudogene)
		TcCLB.507395.30	hypothetical protein
		TcCLB.506717.80	trans-sialidase, Group VIII, putative
		TcCLB.511877.10	Trypomastigote, Alanine, Serine and Valine rich protein (TASV), subfamily B
		TcCLB.506409.100	mucin TcMUCII, putative
		TcCLB.508041.10	SKP1-like protein
		TcCLB.464807.10	serine/threonine protein kinase, putative
		TcCLB.508537.5	tyrosine aminotransferase, putative (fragment)
		TcCLB.511313.10	hypothetical protein, conserved
		TcCLB.506501.100	hypothetical protein
		TcCLB.505025.130	Mucin-associated surface protein (MASP), subgroup S105
		TcCLB.506887.10	trans-sialidase, putative
		TcCLB.507211.20	NEK family Serine/threonine-protein kinase, putative
		TcCLB.439803.9	trans-sialidase (pseudogene), putative
		TcCLB.507485.30	Amastin surface glycoprotein, putative
		TcCLB.510561.40	mucin TcMUCII, putative

		TcCLB.506551.10	protein associated with differentiation 8, putative
		TcCLB.511127.180	hypothetical protein, conserved
		TcCLB.480785.10	serine/threonine kinase, putative
		TcCLB.511875.9	Mucin-associated surface protein (MASP), subgroup S127
		TcCLB.508157.10	serine-alanine-and proline-rich protein (pseudogene), putative
		TcCLB.510371.80	hypothetical protein
		TcCLB.507997.14	trans-sialidase, putative (fragment)
		TcCLB.503903.79	hypothetical protein
		TcCLB.503783.34	mucin-associated surface protein (MASP, pseudogene), putative
		TcCLB.511801.50	tRNA threonylcarbamoyl adenosine modification protein, Sua5/YciO/YrdC/YwlC family, putative
		TcCLB.509007.80	hypothetical protein, conserved
		TcCLB.510275.255	hypothetical protein
		TcCLB.511431.20	hypothetical protein, conserved
		TcCLB.504125.80	hypothetical protein, conserved
		TcCLB.504075.6	kinetoplastid-specific dual specificity phosphatase, putative
		TcCLB.510737.30	mitochondrial carrier protein, putative
		TcCLB.508541.110	Mucin-associated surface protein (MASP), subgroup S068
		TcCLB.506763.210	hypothetical protein
		TcCLB.504125.100	sugar transporter, putative, frameshift
		TcCLB.506341.50	trans-sialidase, Group VIII, putative
		TcCLB.511585.10	hypothetical protein, conserved
		TcCLB.503783.50	surface protease GP63 (pseudogene), putative
		TcCLB.437805.9	trans-sialidase (pseudogene), putative
		TcCLB.509815.90	retrotransposon hot spot protein (RHS, pseudogene), putative
		TcCLB.510431.250	phosphoribosylpyrophosphate synthetase, putative
		TcCLB.511577.120	hypothetical protein, conserved
		TcCLB.506759.90	trans-sialidase (pseudogene), putative
		TcCLB.508647.30	hypothetical protein, conserved
		TcCLB.504051.40	hypothetical protein, conserved
		TcCLB.504427.230	trans-sialidase, Group VIII, putative
		TcCLB.508563.20	trans-sialidase, Group II, putative
		TcCLB.507711.100	Surface membrane protein

		TcCLB.511469.60	transporter, putative
		TcCLB.511213.30	surface protease GP63 (pseudogene), putative
		TcCLB.507831.30	hypothetical protein, conserved
		TcCLB.508265.70	hypothetical protein, conserved
		TcCLB.506289.70	hypothetical protein, conserved
		TcCLB.511587.90	trans-sialidase, Group VIII, putative
		TcCLB.507479.70	trans-sialidase, Group IV, putative
		TcCLB.508675.9	CMGC/DYRK protein kinase, putative (fragment)
		TcCLB.504147.110	asparaginase, putative
		TcCLB.503551.20	hypothetical protein, conserved
		TcCLB.506287.90	chaperone protein DnaJ, putative
		TcCLB.509699.220	trans-sialidase (pseudogene), putative
		TcCLB.506885.180	hypothetical protein, conserved
		TcCLB.509097.20	trans-sialidase, Group V, putative
		TcCLB.509767.140	hypothetical protein
		TcCLB.427247.10	CMGC/DYRK protein kinase, putative
		TcCLB.506945.210	Zinc finger CCCH domain-containing protein 47
		TcCLB.504101.40	trans-sialidase (pseudogene), putative
		TcCLB.511735.34	Eukaryotic protein of unknown function (DUF872), putative
		TcCLB.507953.100	trans-sialidase, Group V, putative
		TcCLB.508137.40	hypothetical protein, conserved
		TcCLB.505945.90	folate/pteridine transporter, putative
		TcCLB.506661.40	zinc finger domain, LSD1 subclass, putative
		TcCLB.504153.50	ribosomal RNA large subunit alpha, 5' & 3' partial
		TcCLB.510209.9	trans-sialidase, putative
		TcCLB.510451.10	trans-sialidase (pseudogene), putative
		TcCLB.510077.20	trans-sialidase (pseudogene), putative
		TcCLB.510769.170	trans-sialidase (pseudogene), putative
		TcCLB.507035.80	trans-sialidase (pseudogene), putative
		TcCLB.508653.20	ribosomal RNA small subunit, 5' partial
		TcCLB.503779.30	hypothetical protein, conserved
		TcCLB.508383.50	C/D small nucleolar RNA (snoRNA), TB10C3C5

		TcCLB.507629.39	hypothetical protein, conserved (fragment)
		TcCLB.511411.30	arginine permease
		TcCLB.507957.140	trans-sialidase (pseudogene), putative
		TcCLB.510013.200	serine-alanine-and proline-rich protein, putative
		TcCLB.410943.10	hypothetical protein, conserved
		TcCLB.509805.210	hypothetical protein, conserved
		TcCLB.511141.30	protein kinase, putative
		TcCLB.508977.80	trans-sialidase, Group V, putative
		TcCLB.508807.5	glycosylphosphatidylinositol-specific phospholipase C, putative
		TcCLB.506895.5	hypothetical protein
		TcCLB.508211.10	oxysterol-binding protein, putative
		TcCLB.510553.20	trans-sialidase (pseudogene), putative
		TcCLB.506759.190	surface protease GP63 (pseudogene), putative
		TcCLB.508239.20	hypothetical protein, conserved
		TcCLB.508383.40	C/D small nucleolar RNA (snoRNA), TB10C3C4
28.14	7	TcCLB.511603.160	Mucin-associated surface protein (MASP), subgroup S082
		TcCLB.510741.202	C/D small nucleolar RNA (snoRNA), TB9C4C2
		TcCLB.511213.60	Mucin-associated surface protein (MASP), subgroup S129
		TcCLB.506147.14	C/D small nucleolar RNA (snoRNA), TB9C2C1
		TcCLB.506357.19	C/D small nucleolar RNA (snoRNA), TB10C4C3
		TcCLB.506825.10	Double RNA binding domain protein 12
		TcCLB.508781.15	H/ACA snoRNA, TC6C1H3
28.21	5	TcCLB.508607.70	trans-sialidase (pseudogene), putative
		TcCLB.508123.10	hypothetical protein, conserved
		TcCLB.506799.110	hypothetical protein
		TcCLB.511173.24	Mucin-associated surface protein (MASP), subgroup S043
		TcCLB.511871.120	Mucin-associated surface protein (MASP)

DOWNREGULATED			
Day comparison	n	Gene ID	Gene name
14.7 21.7 28.14 28.7	1	TcCLB.506893.100	UMP-CMP kinase, mitochondrial, putative
14.7 21.7 28.7	68	TcCLB.508321.21	histone H2A, putative

		TcCLB.509551.30	mitochondrial phosphate transporter, putative
		TcCLB.506223.40	PSP1 C-terminal conserved region, putative
		TcCLB.504089.70	hypothetical protein, conserved
		TcCLB.510525.90	histone H2A, putative
		TcCLB.510509.50	mitochondrial RNA binding protein, putative
		TcCLB.509197.39	cation transporter, putative
		TcCLB.503991.39	2,4-dihydroxyhept-2-ene-1,7-dioic acid aldolase, putative
		TcCLB.511589.200	S-adenosylhomocysteine hydrolase, putative
		TcCLB.507993.330	mevalonate-diphosphate decarboxylase, putative
		TcCLB.510259.50	rotamase, putative
		TcCLB.510101.140	pyruvate phosphate dikinase, putative
		TcCLB.504051.49	hypothetical protein, conserved
		TcCLB.510101.40	60S ribosomal protein L28, putative
		TcCLB.511417.70	Histone-lysine N-methyltransferase, H3 lysine-76 specific
		TcCLB.506287.209	DNA ligase, putative
		TcCLB.507105.50	hypothetical protein
		TcCLB.509069.30	tubulin binding cofactor A-like protein, putative
		TcCLB.504769.80	Nucleoporin NUP92
		TcCLB.511555.80	ribonucleoside-diphosphate reductase small chain, putative
		TcCLB.507809.39	hypothetical protein, conserved
		TcCLB.507083.40	hypothetical protein, conserved
		TcCLB.506289.30	hypothetical protein, conserved
		TcCLB.511135.20	hypothetical protein, conserved
		TcCLB.506567.110	hypothetical protein, conserved
		TcCLB.507709.120	hypothetical protein, conserved
		TcCLB.510317.30	poly(A) polymerase, putative
		TcCLB.510347.29	hypothetical protein, conserved
		TcCLB.507817.18	histone H3, putative
		TcCLB.507837.20	protein kinase, putative
		TcCLB.510535.100	cysteine peptidase C (CPC), putative
		TcCLB.503639.10	inositol-3-phosphate synthase, putative
		TcCLB.509147.50	mucin TcSMUGL, putative

		TcCLB.503831.40	mitochondrial DNA primase, putative
		TcCLB.503697.140	hypothetical protein
		TcCLB.511753.60	ESAG8-associated protein, putative
		TcCLB.506937.10	cytosolic malate dehydrogenase, putative
		TcCLB.506221.110	chromosomal passenger complex 2
		TcCLB.509445.10	tryparedoxin peroxidase, putative
		TcCLB.510525.80	histone H2A, putative
		TcCLB.506503.80	kinesin, putative
		TcCLB.506775.90	PIF1 helicase-like protein, putative
		TcCLB.508675.29	calpain-like cysteine peptidase, putative
		TcCLB.506717.200	hypothetical protein, conserved
		TcCLB.510773.20	Vacuolar proton pyrophosphatase 1, putative
		TcCLB.511047.40	NADH-cytochrome b5 reductase, putative
		TcCLB.511529.80	kinetoplast-associated protein 3
		TcCLB.505989.110	amidinotransferase, putative
		TcCLB.511415.11	retrotransposon hot spot (RHS) protein, putative (fragment)
		TcCLB.506357.40	Elongation factor Tu, mitochondrial, putative
		TcCLB.430737.20	hypothetical protein, conserved (fragment)
		TcCLB.510743.70	lactoylglutathione lyase-like protein, putative
		TcCLB.509245.29	hypothetical protein, conserved
		TcCLB.509201.15	ribosomal protein S29, putative
		TcCLB.511039.10	kinetoplast DNA-associated protein, putative
		TcCLB.506213.50	prostaglandin F synthase (pseudogene), putative
		TcCLB.503841.10	proteasome activator protein pa26, putative
		TcCLB.506735.10	mitochondrial processing peptidase alpha subunit, putative
		TcCLB.458015.4	hypothetical protein, conserved
		TcCLB.509157.70	hypothetical protein, conserved
		TcCLB.506533.142	mucin TcSMUGL, putative
		TcCLB.506925.300	cyclophilin a, putative
		TcCLB.508265.10	glutathione-S-transferase/glutaredoxin, putative
		TcCLB.507929.20	co-chaperone GrpE, putative
		TcCLB.503677.10	phosphomannose isomerase, putative

		TcCLB.509965.290	p22 protein precursor, putative
		TcCLB.508719.30	hypothetical protein, conserved
		TcCLB.506239.10	hypothetical protein, conserved
21.7 28.14 28.7	3	TcCLB.461515.10	mucin TcMUCI, putative (fragment)
		TcCLB.511685.30	mucin TcSMUGS, putative
		TcCLB.510351.31	histone H4, putative
28.14 28.21 28.7	18	TcCLB.503665.21	elongation factor 1-gamma (EF-1-gamma, pseudogene), putative
		TcCLB.420091.10	dispersed gene family protein 1 (DGF-1), putative
		TcCLB.507975.40	dispersed gene family protein 1 (DGF-1), putative
		TcCLB.509247.60	Temperature dependent protein affecting M2 dsRNA replication, putative
		TcCLB.508831.4	dynein heavy chain, putative
		TcCLB.509103.39	dynein heavy chain, putative
		TcCLB.511867.209	dynein heavy chain, putative (fragment)
		TcCLB.504133.15	hypothetical protein, conserved (fragment)
		TcCLB.511751.14	hypothetical protein, conserved (fragment)
		TcCLB.509583.19	dynein heavy chain, putative (fragment)
		TcCLB.506973.70	dispersed gene family protein 1 (DGF-1), putative
		TcCLB.484311.10	calpain-like cysteine peptidase, putative (fragment)
		TcCLB.441061.9	dynein heavy chain, putative (fragment)
		TcCLB.509921.9	dispersed gene family protein 1 (DGF-1), putative
		TcCLB.506849.20	dynein heavy chain (pseudogene), putative
		TcCLB.510531.109	dynein heavy chain, cytosolic, putative (fragment)
		TcCLB.510271.20	dispersed gene family protein 1 (DGF-1, pseudogene), putative
		TcCLB.510913.10	dispersed gene family protein 1 (DGF-1), putative (fragment)
14.7 21.7	3	TcCLB.511001.240	proteasome activator protein pa26, putative
		TcCLB.503893.30	hypothetical protein, conserved
		TcCLB.508209.100	10 kDa heat shock protein, putative
14.7 28.7	6	TcCLB.506925.120	eukaryotic translation initiation factor 5A
		TcCLB.507537.10	cysteine peptidase, clan CA, family C1 (pseudogene), putative
		TcCLB.504427.64	hypothetical protein, conserved
		TcCLB.507483.50	calmodulin
		TcCLB.507639.10	poly-zinc finger protein 2, putative

		TcCLB.503989.10	zinc finger protein 2, putative
21.7 28.7	40	TcCLB.511133.20	thymidine kinase, putative
		TcCLB.511179.130	hypothetical protein, conserved
		TcCLB.442383.19	hypothetical protein
		TcCLB.506683.30	hypothetical protein, conserved
		TcCLB.510603.80	protein tyrosine phosphatase-like protein, putative
		TcCLB.506533.106	mucin TcSMUGL, putative
		TcCLB.510603.60	protein tyrosine phosphatase-like protein, putative
		TcCLB.509129.10	Mitochondrial outer membrane protein porin, putative
		TcCLB.508785.4	Mitochondrial ATP synthase subunit, putative
		TcCLB.508647.200	triosephosphate isomerase
		TcCLB.507979.4	histone H2A variant Z (fragment)
		TcCLB.510181.9	inositol-3-phosphate synthase, putative (fragment)
		TcCLB.511679.10	mucin TcSMUGS, putative
		TcCLB.503599.50	hypothetical protein, conserved
		TcCLB.511751.160	hypothetical protein, conserved
		TcCLB.503955.20	mitochondrial DNA polymerase beta, putative
		TcCLB.510091.110	mitochondrial RNA binding complex 1 subunit, putative
		TcCLB.504427.240	metallo-beta-lactamase-like protein, putative
		TcCLB.482471.10	Mitochondrial ATP synthase subunit, putative
		TcCLB.510349.50	CDP-diacylglycerol--inositol 3-phosphatidyltransferase, putative
		TcCLB.510123.10	hypothetical protein, conserved
		TcCLB.503557.30	hypothetical protein, conserved
		TcCLB.506811.210	cell division cycle protein 45 (CDC45), putative
		TcCLB.511685.10	mucin TcSMUGS, putative
		TcCLB.506401.160	kinetoplastid kinetochore protein 9
		TcCLB.509589.20	squalene monooxygenase, putative
		TcCLB.508839.80	amidase, putative
		TcCLB.506211.160	Mitochondrial ADP/ATP carrier protein 5, putative
		TcCLB.510533.80	ChaC-like protein, putative
		TcCLB.509141.40	Mitochondrial outer membrane protein porin, putative
		TcCLB.506513.80	hypothetical protein, conserved

		TcCLB.510145.20	cofilin/actin depolymerizing factor, putative
		TcCLB.508093.29	hypothetical protein, conserved (pseudogene)
		TcCLB.507875.20	glutamate dehydrogenase, putative
		TcCLB.511871.30	2OG-Fe(II) oxygenase superfamily, putative
		TcCLB.511911.94	hypothetical protein, conserved
		TcCLB.505945.10	DNA repair and recombination helicase protein PIF5, putative
		TcCLB.507611.270	hypothetical protein, conserved
		TcCLB.506825.190	hypothetical protein
		TcCLB.506289.9	DNA ligase, putative (fragment)
28.14 28.7	9	TcCLB.509141.20	hypothetical protein
		TcCLB.504557.20	UDP-Gal or UDP-GlcNAc-dependent glycosyltransferase, putative
		TcCLB.504557.50	dispersed gene family protein 1 (DGF-1), putative
		TcCLB.507865.9	dispersed gene family protein 1 (DGF-1), putative
		TcCLB.506411.10	thimet oligopeptidase, putative
		TcCLB.511677.10	dispersed gene family protein 1 (DGF-1), putative
		TcCLB.511685.20	mucin TcSMUGS, putative
		TcCLB.474341.9	dynein heavy chain, putative
		TcCLB.509979.150	nitrilase, putative
28.21 28.7	17	TcCLB.507719.9	dispersed gene family protein 1 (DGF-1), putative
		TcCLB.509173.9	hypothetical protein, conserved
		TcCLB.506097.9	dynein heavy chain, putative (fragment)
		TcCLB.405179.10	dynein heavy chain, putative (fragment)
		TcCLB.510815.9	dispersed gene family protein 1 (DGF-1), putative
		TcCLB.507213.60	retrotransposon hot spot protein (RHS, pseudogene), putative
		TcCLB.508829.9	dynein heavy chain, putative (fragment)
		TcCLB.508803.19	dispersed gene family protein 1 (DGF-1), putative
		TcCLB.509599.20	N-terminal region of Chorein, a TM vesicle-mediated sorter, putative
		TcCLB.511869.9	dynein heavy chain, putative (fragment)
		TcCLB.441241.10	microtubule-associated protein Gb4, putative (fragment)
		TcCLB.511171.10	dispersed gene family protein 1 (DGF-1), putative
		TcCLB.507531.19	phosphatidylinositol 4-kinase alpha, putative
		TcCLB.506925.550	cysteine peptidase, Clan CA, family C2, putative, frameshift

		TcCLB.448567.9	dynein heavy chain, putative
		TcCLB.504133.45	Temperature dependent protein affecting M2 dsRNA replication, putative
		TcCLB.510509.9	inositol 1,4,5-trisphosphate receptor, putative (fragment)
28.14 28.21	1	TcCLB.507477.10	dispersed gene family protein 1 (DGF-1), putative
14.7	12	TcCLB.510911.40	hypothetical protein, conserved
		TcCLB.507283.39	trans-sialidase (pseudogene), putative
		TcCLB.509551.70	Co-chaperone protein P23
		TcCLB.510741.202	C/D small nucleolar RNA (snoRNA), TB9C4C2
		TcCLB.511213.60	Mucin-associated surface protein (MASP), subgroup S129
		TcCLB.509011.40	calreticulin, putative
		TcCLB.506357.19	C/D small nucleolar RNA (snoRNA), TB10C4C3
		TcCLB.507641.240	hypothetical protein
		TcCLB.506465.14	hypothetical protein, conserved
		TcCLB.507035.75	retrotransposon hot spot protein (RHS, pseudogene), putative
		TcCLB.506933.60	mitochondrial RNA binding protein, putative
		TcCLB.508647.280	malic enzyme
21.7	26	TcCLB.509979.119	hypothetical protein, conserved
		TcCLB.506795.90	'Cold-shock' DNA-binding domain containing protein, putative
		TcCLB.508209.120	10 kDa heat shock protein, putative
		TcCLB.507711.20	paraflagellar rod component, putative
		TcCLB.511311.45	C/D small nucleolar RNA (snoRNA), TB5C1C1
		TcCLB.511805.15	ribosomal protein S29, putative
		TcCLB.511365.80	mitochondrial carrier protein, putative
		TcCLB.508123.10	hypothetical protein, conserved
		TcCLB.506885.14	ribosomal protein L37, putative
		TcCLB.424195.5	hypothetical protein, conserved
		TcCLB.509081.10	elongation factor 1-gamma (EF-1-gamma), putative
		TcCLB.511737.9	Translation machinery associated TMA7, putative
		TcCLB.510755.98	kinetoplastid membrane protein 11
		TcCLB.511369.30	elongation factor 1-alpha, putative
		TcCLB.510101.180	40S ribosomal protein S5, putative
		TcCLB.510719.30	ribosomal protein L29, putative

		TcCLB.506165.16	protein kinase, putative
		TcCLB.506691.64	cytochrome c oxidase copper chaperone, putative
		TcCLB.505931.10	surface protease GP63, putative
		TcCLB.511355.24	hypothetical protein, conserved
		TcCLB.506517.149	Acyl CoA binding protein, putative
		TcCLB.511687.19	Mitochondrial outer membrane protein porin, putative (fragment)
		TcCLB.441401.10	imidazolonepropienase, putative
		TcCLB.504041.4	RNA polymerase I
		TcCLB.504005.54	hypothetical protein, conserved
		TcCLB.506503.170	hypothetical protein, conserved
28.7	166	TcCLB.506857.20	pyrroline-5-carboxylate reductase, putative
		TcCLB.509157.80	DnaJ homolog, putative
		TcCLB.511217.209	hypothetical protein, conserved (fragment)
		TcCLB.506647.20	hypothetical protein, conserved
		TcCLB.510609.20	hypothetical protein, conserved
		TcCLB.506847.4	hypothetical protein, conserved
		TcCLB.506945.240	ATP synthase, epsilon chain, putative
		TcCLB.503577.9	delta-1-pyrroline-5-carboxylate dehydrogenase (pseudogene), putative
		TcCLB.506739.30	hypothetical protein
		TcCLB.506563.70	unspecified product
		TcCLB.511559.50	hypothetical protein, conserved
		TcCLB.511303.34	Macro domain containing protein, putative
		TcCLB.507595.10	dispersed gene family protein 1 (DGF-1, pseudogene), putative
		TcCLB.506795.44	Bifunctional NAD(P)H-hydrate repair enzyme
		TcCLB.506351.20	hypothetical protein, conserved
		TcCLB.510493.4	hypothetical protein, conserved (fragment)
		TcCLB.509965.20	hypothetical protein
		TcCLB.506525.45	hypothetical protein, conserved (pseudogene)
		TcCLB.503895.19	hypothetical protein
		TcCLB.506649.20	P-type H+ATPase, putative
		TcCLB.507445.50	myosin heavy chain, putative
		TcCLB.509153.90	dihydrofolate reductase-thymidylate synthase

		TcCLB.506925.360	hypothetical protein
		TcCLB.506529.120	hypothetical protein
		TcCLB.426397.9	kinesin, putative (fragment)
		TcCLB.511875.29	mucin-associated surface protein (MASP, pseudogene), putative
		TcCLB.506885.340	Mitochondrial import receptor subunit ATOM40, putative
		TcCLB.507533.10	phosphatidylinositol 4-kinase alpha, putative
		TcCLB.506135.70	hypothetical protein, conserved
		TcCLB.507093.229	U-rich RNA-binding protein UBP-2
		TcCLB.504153.120	hypothetical protein, conserved
		TcCLB.511125.9	dispersed gene family protein 1 (DGF-1), putative
		TcCLB.507011.30	CRAL/TRIO domain containing protein, putative
		TcCLB.511733.130	chaperonin HSP60, putative (fragment)
		TcCLB.437545.10	3-oxo-5-alpha-steroid 4-dehydrogenase, putative
		TcCLB.511529.210	hypothetical protein, conserved
		TcCLB.506931.70	hypothetical protein, conserved
		TcCLB.509157.220	hypothetical protein, conserved
		TcCLB.508727.50	hypothetical protein, conserved
		TcCLB.510877.30	hypothetical protein, conserved
		TcCLB.509825.14	40S ribosomal protein SA, putative
		TcCLB.508717.36	selenophosphate synthetase, putative
		TcCLB.511729.60	MORN repeat-containing protein 1
		TcCLB.510533.10	Cytoplasmic dynein 1 heavy chain (DYNC1H1), putative
		TcCLB.511681.20	histone H4, putative
		TcCLB.511363.4	hypothetical protein, conserved
		TcCLB.509805.260	dispersed gene family protein 1 (DGF-1, pseudogene), putative
		TcCLB.506227.190	hypothetical protein
		TcCLB.503799.4	aurora B kinase, putative (fragment)
		TcCLB.506925.490	kinetoplastid kinetochore protein 7
		TcCLB.507089.260	cyclin 6, putative
		TcCLB.509233.180	ATP synthase subunit beta, mitochondrial, putative
		TcCLB.506239.40	U-box domain containing protein, putative
		TcCLB.510955.20	hypothetical protein, conserved

		TcCLB.508813.15	heat shock protein 70, putative (fragment)
		TcCLB.503637.30	serine peptidase, Clan SC, Family S9B
		TcCLB.510091.80	pyruvate dehydrogenase E1 beta subunit, putative
		TcCLB.508147.159	dispersed gene family protein 1 (DGF-1), putative
		TcCLB.511217.120	peptidyl-prolyl cis-trans isomerase, putative
		TcCLB.511691.39	hypothetical protein, conserved (fragment)
		TcCLB.507093.220	RNA-binding protein UBP1, putative
		TcCLB.506933.20	ribonucleoside-diphosphate reductase large chain, putative
		TcCLB.508717.20	NADH dehydrogenase, putative
		TcCLB.511115.30	hypothetical protein, conserved
		TcCLB.473633.9	dispersed gene family protein 1 (DGF-1), putative (fragment)
		TcCLB.506405.50	serine-palmitoyl-CoA transferase, putative
		TcCLB.509803.70	dispersed gene family protein 1 (DGF-1, pseudogene), putative
		TcCLB.511693.10	hypothetical protein
		TcCLB.511181.150	hypothetical protein, conserved
		TcCLB.506127.30	phenylalanyl-tRNA synthetase, putative
		TcCLB.506967.20	hypothetical protein
		TcCLB.504147.70	hypothetical protein, conserved
		TcCLB.510101.320	hypothetical protein
		TcCLB.506167.20	STAG domain containing protein, putative
		TcCLB.510105.240	short chain 3-hydroxyacyl-CoA dehydrogenase, putative
		TcCLB.511589.60	hypothetical protein, conserved
		TcCLB.509199.24	ribose 5-phosphate isomerase type B
		TcCLB.405165.19	hypothetical protein, conserved (fragment)
		TcCLB.511749.9	hypothetical protein, conserved (fragment)
		TcCLB.508325.30	dispersed gene family protein 1 (DGF-1), putative
		TcCLB.507929.10	kinesin, putative (fragment)
		TcCLB.506947.110	myosin heavy chain, putative, frameshift
		TcCLB.511211.30	hypothetical protein
		TcCLB.510879.180	hypothetical protein
		TcCLB.509141.30	hypothetical protein
		TcCLB.508715.10	oxidoreductase, putative (fragment)

		TcCLB.506265.130	hypothetical protein, conserved
		TcCLB.510353.10	major vault protein, putative
		TcCLB.508567.100	hypothetical protein, conserved
		TcCLB.510127.79	actin 3, putative (fragment)
		TcCLB.506367.30	1,2-Dihydroxy-3-keto-5-methylthiopentene dioxygenase, putative
		TcCLB.511211.120	receptor for activated C kinase 1, putative
		TcCLB.505807.60	hypothetical protein, conserved
		TcCLB.507483.30	calmodulin, putative
		TcCLB.509245.10	hypothetical protein, conserved
		TcCLB.509967.189	kinesin, putative (fragment)
		TcCLB.413977.10	guide RNA associated protein, GAP1, putative
		TcCLB.509625.30	Protein of unknown function (DUF1014), putative
		TcCLB.511365.4	hypothetical protein, conserved (fragment)
		TcCLB.506951.10	retrotransposon hot spot protein (RHS, pseudogene), putative
		TcCLB.511211.130	receptor for activated C kinase 1, putative
		TcCLB.508769.10	hypothetical protein, conserved (pseudogene)
		TcCLB.511001.40	hypothetical protein
		TcCLB.511545.90	hypothetical protein
		TcCLB.504423.30	hypothetical protein, conserved
		TcCLB.506925.150	protein phosphatase 2C, putative
		TcCLB.509171.70	hypothetical protein, conserved
		TcCLB.508217.9	Inner arm dynein 5-1
		TcCLB.508163.390	hypothetical protein, conserved
		TcCLB.506533.34	phosphatidylinositol kinase related protein, putative
		TcCLB.503757.10	40S ribosomal protein SA, putative
		TcCLB.510535.20	hypothetical protein, conserved
		TcCLB.508265.100	cytoskeleton associated protein, putative
		TcCLB.510747.60	cytochrome P450 reductase, putative
		TcCLB.508387.160	Phosphatidylinositol 4-phosphate 5-kinase, putative
		TcCLB.503897.20	hypothetical protein, conserved
		TcCLB.511071.50	3-hydroxy-3-methylglutaryl-CoA synthase, putative
		TcCLB.503419.50	MRB1-associated protein, putative

		TcCLB.509233.150	cytochrome oxidase subunit VII
		TcCLB.511539.9	hypothetical protein, conserved
		TcCLB.510105.50	fibrillarin, putative
		TcCLB.510311.170	hypothetical protein, conserved
		TcCLB.510285.80	hypothetical protein, conserved
		TcCLB.506563.10	pumilio/PUF RNA binding protein 9, putative
		TcCLB.509067.40	NADH dehydrogenase subunit NI8M, putative
		TcCLB.506649.90	hypothetical protein, conserved
		TcCLB.509099.110	hypothetical protein, conserved
		TcCLB.507867.100	hypothetical protein
		TcCLB.504089.50	Flagellar radial spoke protein 4/6
		TcCLB.506753.130	rhodanese-like domain containing protein, putative
		TcCLB.507039.10	iron superoxide dismutase, putative
		TcCLB.508387.20	methylthioadenosine phosphorylase, putative
		TcCLB.507037.80	hypothetical protein, conserved
		TcCLB.506859.70	guide RNA associated protein, GAP2, putative
		TcCLB.506691.14	ATP-dependent DEAD/H RNA helicase, putative (fragment)
		TcCLB.507795.60	tryptophanyl-tRNA synthetase, putative, frameshift
		TcCLB.506163.50	hypothetical protein, conserved
		TcCLB.511421.200	ARM-like helical domain-containing protein
		TcCLB.509585.10	dynein heavy chain, putative
		TcCLB.506529.669	Eukaryotic protein of unknown function (DUF866), putative
		TcCLB.509099.30	paraflagellar rod protein 5, putative
		TcCLB.511445.20	radial spoke protein 3, putative
		TcCLB.508271.10	phenylalanyl-tRNA synthetase, putative
		TcCLB.506775.80	14-3-3 protein 2, putative
		TcCLB.507641.190	kinetoplastid kinetochore protein 1, putative
		TcCLB.506181.104	actyltransferase-like protein
		TcCLB.508281.9	dispersed gene family protein 1 (DGF-1), putative
		TcCLB.436521.9	mevalonate kinase, putative (fragment)
		TcCLB.508955.20	hypothetical protein, conserved
		TcCLB.504625.70	kinetoplast DNA-associated protein, putative

		TcCLB.506543.20	ATP-grasp domain containing protein, putative
		TcCLB.511537.69	hypothetical protein, conserved
		TcCLB.510755.120	RNA-binding protein, putative
		TcCLB.508569.70	hypothetical protein, conserved
		TcCLB.506529.410	primase 2, putative
		TcCLB.506749.30	Flagellar calcium-binding protein (fragment)
		TcCLB.509831.36	guide RNA-binding protein of 21 kDa
		TcCLB.510847.70	dispersed gene family protein 1 (DGF-1), putative
		TcCLB.504137.80	adenylyl cyclase-associated protein, putative
		TcCLB.507603.270	major cysteine proteinase, putative
		TcCLB.510507.20	3-ketoacyl-CoA thiolase, putative
		TcCLB.510101.30	60S ribosomal protein L28, putative
		TcCLB.507999.10	dispersed gene family protein 1 (DGF-1), putative
		TcCLB.509351.10	delta-1-pyrroline-5-carboxylate dehydrogenase, putative
		TcCLB.508567.60	hypothetical protein
		TcCLB.510595.4	hypothetical protein, conserved
28.14	3	TcCLB.506129.80	retrotransposon hot spot protein (RHS, pseudogene), putative
		TcCLB.506357.50	alcohol dehydrogenase, putative
		TcCLB.509845.9	dispersed gene family protein 1 (DGF-1), putative
28.21	23	TcCLB.508903.90	dispersed gene family protein 1 (DGF-1, pseudogene), putative
		TcCLB.506213.120	ATP-dependent RNA helicase HEL67
		TcCLB.511371.10	retrotransposon hot spot (RHS) protein, putative
		TcCLB.507827.10	dispersed gene family protein 1 (DGF-1), putative
		TcCLB.511797.220	dispersed gene family protein 1 (DGF-1), putative
		TcCLB.509891.70	SPRY domain/HECT-domain (ubiquitin-transferase), putative
		TcCLB.510855.4	hypothetical protein, conserved
		TcCLB.507871.9	dispersed gene family protein 1 (DGF-1), putative
		TcCLB.510773.9	Thioredoxin-like/Protein of unknown function (DUF3638)/Protein of unknown function (DUF3645), putative
		TcCLB.509919.9	dispersed gene family protein 1 (DGF-1), putative (fragment)
		TcCLB.511435.49	phosphatidylinositol 3-kinase, putative
		TcCLB.508559.29	dispersed gene family protein 1 (DGF-1), putative
		TcCLB.509887.19	dispersed gene family protein 1 (DGF-1), putative

		TcCLB.511443.10	calpain-like cysteine peptidase, putative, frameshift
		TcCLB.509013.19	calpain-like cysteine peptidase, putative (fragment)
		TcCLB.507805.30	hypothetical protein, conserved (pseudogene)
		TcCLB.506409.130	Mucin-associated surface protein (MASP) (pseudogene)
		TcCLB.508831.250	dispersed gene family protein 1 (DGF-1), putative
		TcCLB.508837.34	dispersed gene family protein 1 (DGF-1), putative (fragment)
		TcCLB.508163.70	dispersed gene family protein 1 (DGF-1), putative
		TcCLB.510607.29	dispersed gene family protein 1 (DGF-1), putative (fragment)
		TcCLB.507507.49	hypothetical protein, conserved
		TcCLB.511437.10	surface protease GP63, putative

The total number of differentially expressed genes (n) is indicated for each of the comparison between the cultured days analysed (x.y: dayx vs dayy or Dx vs Dy). Data obtained from.[36]

TABLE II

Differential expression values of surface gene families (*trans-sialidase*, *mucin*, *GP63*, *TASV*, and *amastin*). During the prolonged starvation of *Trypanosoma cruzi* epimastigote culture time points at day 7, corresponding to the exponential phase, day 14, early stationary phase, day 21, intermediate stationary phase and day 28, the final of the stationary phase (D7, D14, D21 and D28 respectively) were selected for analysis. The table shows *fold-change* (FC) and adjusted *p*-values (pAdj) obtained from differential expression analysis at D14, D21, and D28 compared to D7. These data correspond to Fig. 4 and Supplementary data (Fig 3) and highlight the coordinated modulation of multigene families associated with parasite adaptation and persistence within the host

IDs	FC 14vs7	FC 21vs7	FC 28vs7	pAdj 14vs7	pAdj 21vs7	pAdj 28vs7	
TcCLB.505931.30	1.03632834	1.13724964	1.00607991	0.77899008	0.12360502	0.94326482	trans-sialidase, Group I, putative
TcCLB.506961.25	0.97585438	0.95794085	0.81203004	0.85122575	0.60688789	0.01569336	trans-sialidase, Group I, putative
TcCLB.507979.30	0.83152161	0.9128211	0.76048405	0.07579915	0.28581453	0.00195043	trans-sialidase, Group I, putative
TcCLB.508089.10	1.20473833	1.18509606	1.34717758	0.01289512	0.00859922	4.2235E-06	trans-sialidase, Group I, putative
TcCLB.508717.60	0.97096885	1.13354676	0.97949727	0.81896013	0.13171621	0.8065268	trans-sialidase, Group I, putative
TcCLB.509817.50	1.13630581	1.08350093	1.05357169	0.3810493	0.46630234	0.64214112	trans-sialidase, Group I, putative
TcCLB.510055.20	1.18156677	1.58106876	1.54385051	0.39181357	0.0013768	0.00273866	trans-sialidase, Group I, putative
IDs	FC 14vs7	FC 21vs7	FC 28vs7	pAdj 14vs7	pAdj 21vs7	pAdj 28vs7	
TcCLB.504099.50	1.30746978	1.79945133	2.23863792	1.0846E-09	3.4979E-47	2.3103E-87	trans-sialidase, Group II, putative
TcCLB.506021.20	1.27624776	2.22399621	2.71966084	0.13356577	2.7367E-10	2.4857E-15	trans-sialidase, Group II, putative
TcCLB.508607.50	1.61132199	2.10752262	2.03845102	0.00286038	6.8374E-08	3.8292E-07	trans-sialidase, Group II, putative
TcCLB.508563.20	1.50278011	1.9321519	2.349495	0.0046668	1.0987E-07	5.3985E-12	trans-sialidase, Group II, putative
TcCLB.511585.230	2.58384649	3.62371835	3.83476706	0.00068594	1.5208E-07	4.9594E-08	trans-sialidase, Group II, putative
TcCLB.511349.100	1.42509184	1.56936937	2.20613647	0.00203125	7.9293E-06	2.3447E-15	trans-sialidase, Group II, putative
TcCLB.506345.90	2.32485371	2.05006418	3.03266701	4.836E-06	2.4582E-05	4.0845E-11	trans-sialidase, Group II, putative
TcCLB.504769.100	1.41489836	1.98159548	2.03367483	0.07801183	1.6386E-05	9.7604E-06	trans-sialidase, Group II, putative
TcCLB.506129.30	1.79771308	2.74615497	2.70176037	0.05117435	4.1394E-05	6.4356E-05	trans-sialidase, Group II, putative
TcCLB.510005.20	1.26305472	2.01722112	2.07884959	0.31499577	5.1623E-05	2.7791E-05	trans-sialidase, Group II, putative
TcCLB.507879.10	1.29030044	1.64184512	2.8971254	0.51170452	0.07315653	8.5301E-05	trans-sialidase, Group II, putative
TcCLB.504343.10	1.8624436	2.13601054	3.65877461	0.01726806	0.00063552	2.6334E-09	trans-sialidase, Group II, putative
TcCLB.506757.90	3.42513593	3.51452136	5.54963868	0.00313911	0.00060102	2.3022E-06	trans-sialidase, Group II, putative
TcCLB.507907.20	2.68577318	4.71212035	2.78009673	0.10104804	0.00120373	0.04092889	trans-sialidase, Group II, putative
TcCLB.507875.220	1.55607234	2.67844528	1.915296	0.3572048	0.00479327	0.07298384	trans-sialidase, Group II, putative
TcCLB.511311.20	1.60496329	2.20381703	2.1042551	0.16436896	0.00330139	0.00620286	trans-sialidase, Group II, putative
TcCLB.508285.60	1.57389764	2.18943063	2.54648202	0.21977256	0.00610485	0.00108814	trans-sialidase, Group II, putative
TcCLB.507611.170	1.62607487	1.70978847	2.01713545	0.17440678	0.06036612	0.01407594	trans-sialidase, Group II, putative

TcCLB.509739.10	1.70123249	1.69948627	2.55317136	0.02069792	0.0069379	1.437E-06	trans-sialidase, Group II, putative
TcCLB.506455.30	1.89812266	2.12326086	2.62316357	0.06566591	0.00902034	0.00080588	trans-sialidase, Group II, putative
TcCLB.510403.30	1.23719493	1.05198954	2.24977312	0.52549092	0.83659377	0.00054944	trans-sialidase, Group II, putative
TcCLB.507479.20	2.61278092	2.24358139	2.8285464	0.0136388	0.01687225	0.00203718	trans-sialidase, Group II, putative
IDs	FC 14vs7	FC 21vs7	FC 28vs7	pAdj 14vs7	pAdj 21vs7	pAdj 28vs7	
TcCLB.504425.10	1.18291287	1.12695651	0.95727882	0.02411862	0.06051255	0.5026667	trans-sialidase, Group III, putative
TcCLB.506885.210	0.41339013	1.58069717	1.17266414	NA	0.32691838	0.74299341	trans-sialidase, Group III, putative
TcCLB.511129.40	0.67383447	0.52023472	1.11372053	NA	0.32196406	0.8625234	trans-sialidase, Group III, putative
TcCLB.511911.60	1.24024995	1.32156757	1.12891489	0.34295531	0.10453542	0.4911754	trans-sialidase, Group III, putative
IDs	FC 14vs7	FC 21vs7	FC 28vs7	pAdj 14vs7	pAdj 21vs7	pAdj 28vs7	
TcCLB.503447.50	2.28526395	2.74394063	4.47997483	1.5488E-05	4.7223E-09	1.0724E-18	trans-sialidase, Group IV, putative
TcCLB.504341.10	1.3920911	2.20768067	1.96780925	0.22406028	0.00013408	0.00136943	trans-sialidase, Group IV, putative
TcCLB.506129.50	1.36192049	2.13688655	2.1236919	0.22560126	0.00010129	0.00013449	trans-sialidase, Group IV, putative
TcCLB.506951.90	1.97878684	2.29479852	3.98099237	0.01701733	0.0006384	7.1323E-09	trans-sialidase, Group IV, putative
TcCLB.507479.70	1.41987392	1.68166636	2.07030485	0.13933388	0.006055	0.00011683	trans-sialidase, Group IV, putative
TcCLB.508325.230	1.59941397	2.03195352	2.50762631	0.00150478	3.9864E-08	9.6617E-13	trans-sialidase, Group IV, putative
TcCLB.510307.284	3.79176142	3.67756061	4.39160966	0.01370555	0.00521585	0.00150114	trans-sialidase, Group IV, putative
TcCLB.510491.60	1.63828504	2.08063614	2.50745907	0.00019588	5.484E-10	6.7719E-15	trans-sialidase, Group IV, putative
IDs	FC 14vs7	FC 21vs7	FC 28vs7	pAdj 14vs7	pAdj 21vs7	pAdj 28vs7	
TcCLB.503717.10	2.68950554	2.11372119	2.37749395	0.00207691	0.00961845	0.00286422	trans-sialidase, Group V, putative
TcCLB.504239.260	1.38331541	1.84564385	1.88093399	0.03670109	1.8532E-06	1.1549E-06	trans-sialidase, Group V, putative
TcCLB.506757.120	1.12806825	1.46473133	1.52638547	0.53581221	0.00528098	0.00224746	trans-sialidase, Group V, putative
TcCLB.507091.60	1.45581293	1.86830471	2.20345205	0.23033642	0.00962987	0.00107668	trans-sialidase, Group V, putative
TcCLB.507163.70	1.96995075	1.89674733	2.51595621	0.01905597	0.00997267	0.00018462	trans-sialidase, Group V, putative
TcCLB.507953.100	1.5078043	1.91938578	2.01671363	0.03110251	3.8313E-05	1.1563E-05	trans-sialidase, Group V, putative
TcCLB.508109.60	1.57837252	2.39440173	2.21649695	0.21385307	0.00198828	0.00551319	trans-sialidase, Group V, putative
TcCLB.508977.80	1.36191501	1.83216109	2.06032451	0.13645134	0.0002408	1.2532E-05	trans-sialidase, Group V, putative
TcCLB.509699.40	2.5537178	2.54025727	2.52177309	0.01361936	0.00441424	0.00533213	trans-sialidase, Group V, putative
TcCLB.509979.320	1.0121415	1.30356956	1.30990243	0.94400534	0.00875317	0.00830925	trans-sialidase, Group V, putative
TcCLB.510021.120	2.20419309	2.60555142	3.08759853	0.01888429	0.00080028	7.9495E-05	trans-sialidase, Group V, putative
TcCLB.510021.180	1.12054328	1.37706644	1.55376989	0.36612413	0.00063145	2.7336E-06	trans-sialidase, Group V, putative
TcCLB.510025.50	1.21060441	1.6782198	2.01490293	0.2501987	4.9725E-05	3.9752E-08	trans-sialidase, Group V, putative

TcCLB.510205.40	1.75328211	2.1600936	2.5946015	4.1407E-05	3.447E-10	7.5587E-15	trans-sialidase, Group V, putative
TcCLB.510377.10	1.23763089	1.43651151	1.69471526	0.12147296	0.00109061	2.0009E-06	trans-sialidase, Group V, putative
TcCLB.510377.330	1.17902865	1.57184838	1.56272098	0.37957316	0.00097096	0.00135861	trans-sialidase, Group V, putative
TcCLB.511401.90	1.45653464	1.87912934	2.70861945	0.15916163	0.00279982	1.771E-06	trans-sialidase, Group V, putative
TcCLB.511603.450	1.21933911	1.68589775	1.29291875	0.45881387	0.00630548	0.19647393	trans-sialidase, Group V, putative
TcCLB.511625.130	1.66380141	2.43653062	2.87536058	0.12499281	0.00076663	6.6421E-05	trans-sialidase, Group V, putative
IDs	FC 14vs7	FC 21vs7	FC 28vs7	pAdj 14vs7	pAdj 21vs7	pAdj 28vs7	
TcCLB.506409.170	1.28646234	2.05168254	2.29860056	0.38031826	0.00062881	7.8649E-05	trans-sialidase, Group VI, putative
TcCLB.506967.60	1.49696481	0.88882516	2.40615105	0.49141111	0.79241061	0.03582836	trans-sialidase, Group VI, putative
TcCLB.507747.180	1.7158689	2.66739595	2.88121502	0.06228554	2.8949E-05	7.297E-06	trans-sialidase, Group VI, putative
TcCLB.508107.30	1.08371374	1.4459708	1.10330277	0.83736406	0.13403077	0.70161348	trans-sialidase, Group VI, putative
TcCLB.508163.250	0.21802258	0.56424849	0.65372496	NA	0.33282667	0.47289399	trans-sialidase, Group VI, putative
TcCLB.510279.110	1.15962638	1.10525756	1.7506466	0.4909592	0.52393378	0.0002797	trans-sialidase, Group VI, putative
TcCLB.510279.230	0.94390722	1.24827183	1.45535136	0.90028982	0.41935512	0.17256406	trans-sialidase, Group VI, putative
TcCLB.511173.370	0.9791463	1.44702863	2.05264279	0.97129061	0.27153569	0.02991194	trans-sialidase, Group VI, putative
IDs	FC 14vs7	FC 21vs7	FC 28vs7	pAdj 14vs7	pAdj 21vs7	pAdj 28vs7	
TcCLB.507121.20	1.95697407	1.88391844	1.58738469	0.02463225	0.01306635	0.07697858	trans-sialidase, Group VII, putative
TcCLB.507875.70	1.2980942	1.80535097	1.60990873	0.19761054	0.00016726	0.00299683	trans-sialidase, Group VII, putative
TcCLB.508229.60	2.75641483	2.2776117	3.3324838	7.8256E-05	0.00044631	2.0812E-07	trans-sialidase, Group VII, putative
TcCLB.509377.20	1.48454317	1.58590903	1.90041027	0.00012915	6.7874E-07	4.5843E-12	trans-sialidase, Group VII, putative
TcCLB.509581.10	1.20460191	1.86595233	1.55596851	0.67119596	0.03303156	0.14120673	trans-sialidase, Group VII, putative
TcCLB.509843.20	0.9594151	1.32260083	0.99448715	NA	0.62752903	0.99270145	trans-sialidase, Group VII, putative
TcCLB.511875.20	0.47832215	0.87545378	1.02017223	NA	0.79480711	0.96891499	trans-sialidase, Group VII, putative
IDs	FC 14vs7	FC 21vs7	FC 28vs7	pAdj 14vs7	pAdj 21vs7	pAdj 28vs7	
TcCLB.509157.170	1.2963504	1.61501399	1.85701157	0.0067565	5.6835E-09	6.1281E-14	trans-sialidase, Group VIII, putative
TcCLB.504427.230	1.6696519	1.93595525	2.37561211	0.00511788	3.0623E-05	4.456E-08	trans-sialidase, Group VIII, putative
TcCLB.506961.150	1.4284161	2.32111151	1.75773052	0.25391908	0.00036402	0.02056975	trans-sialidase, Group VIII, putative
TcCLB.503907.10	1.40336169	1.47710423	1.80121756	0.00404578	0.00015522	1.1039E-08	trans-sialidase, Group VIII, putative
TcCLB.507555.50	1.70306541	2.06532496	2.77657206	0.13860692	0.01171617	0.00035235	trans-sialidase, Group VIII, putative
TcCLB.506717.80	1.33877047	1.61130384	2.24603552	0.23945701	0.01289676	2.01E-05	trans-sialidase, Group VIII, putative
TcCLB.510847.10	1.40957846	1.70160393	2.44011618	0.22268924	0.01492439	3.4469E-05	trans-sialidase, Group VIII, putative
TcCLB.508515.150	1.40652906	1.90460799	1.53434313	0.47921415	0.06196109	0.2261647	trans-sialidase, Group VIII, putative

TcCLB.510971.30	4.03371035	4.76595076	9.69721803	NA	0.06355106	0.00612107	trans-sialidase, Group VIII, putative
TcCLB.510307.240	1.29288814	1.69585698	1.59064897	0.56202049	0.08794255	0.1410285	trans-sialidase, Group VIII, putative
TcCLB.507759.10	1.36046335	1.79411389	1.87490243	0.54275203	0.10084383	0.08019838	trans-sialidase, Group VIII, putative
TcCLB.504769.140	1.35037699	1.29136815	1.49574293	0.17063255	0.14710377	0.022723	trans-sialidase, Group VIII, putative
TcCLB.511587.90	2.47893562	1.92844163	2.6510016	0.07591524	0.12701199	0.02224044	trans-sialidase, Group VIII, putative
TcCLB.506341.50	3.50262576	2.47470982	5.41496374	NA	0.14304833	0.00472166	trans-sialidase, Group VIII, putative
TcCLB.510125.20	1.51811112	1.42522271	2.35481073	0.31636394	0.2695718	0.00633919	trans-sialidase, Group VIII, putative
TcCLB.509765.50	1.37666511	1.37938128	1.20304483	0.4879871	0.33769784	0.59019011	trans-sialidase, Group VIII, putative
TcCLB.505365.60	1.06978875	1.2059902	1.74034278	0.88985508	0.53256648	0.06098786	trans-sialidase, Group VIII, putative
TcCLB.506683.110	1.22822889	1.03326886	1.57141482	0.64668848	0.91723403	0.14462126	trans-sialidase, Group VIII, putative
IDs	FC 14vs7	FC 21vs7	FC 28vs7	pAdj 14vs7	pAdj 21vs7	pAdj 28vs7	
TcCLB.506533.106	0.57171913	0.46861169	0.43147711	0.00495611	1.3778E-05	1.52E-06	mucin TcSMUGL, putative
TcCLB.506533.142	0.40027141	0.4148359	0.34766078	6.6941E-22	4.7397E-22	6.1378E-30	mucin TcSMUGL, putative
TcCLB.509147.50	0.41168226	0.40957936	0.30417058	0.0020951	0.00047562	1.3926E-05	mucin TcSMUGL, putative
TcCLB.511679.10	0.50372039	0.41708706	0.36333732	1.0625E-13	4.1574E-23	3.2158E-29	mucin TcSMUGL, putative
TcCLB.511685.10	0.6253335	0.46210319	0.44530635	0.01062455	1.2789E-06	4.1159E-07	mucin TcSMUGL, putative
TcCLB.511685.20	0.61950691	0.49273067	0.21427737	0.23705383	0.02645394	2.5712E-06	mucin TcSMUGL, putative
TcCLB.511685.30	0.61788969	0.37862246	0.25527384	0.2320717	0.00233559	2.4905E-05	mucin TcSMUGL, putative
IDs	FC 14vs7	FC 21vs7	FC 28vs7	pAdj 14vs7	pAdj 21vs7	pAdj 28vs7	
TcCLB.503665.40	1.64536929	2.24568222	3.54907063	0.07972918	0.00041597	1.8239E-08	
TcCLB.504039.250	1.71901212	17.1660697	29.7524417	0.5712539	2.4266E-06	1.5678E-08	
TcCLB.505931.10	1.13910111	0.2458792	1.01448948	0.84322559	0.00751108	0.97354291	
TcCLB.506289.170	1.50744188	2.03166722	1.89301188	0.09341302	0.00032715	0.00145995	
TcCLB.506599.250	1.11741525	1.9977183	1.71242663	0.79179377	0.00782706	0.04441351	
TcCLB.506779.180	1.31665329	1.50680204	1.62249897	0.01669022	2.9772E-05	9.0141E-07	
TcCLB.506971.20	2.24338918	3.79818368	5.35731286	1.0158E-16	2.8137E-49	8.7109E-78	
TcCLB.507747.10	1.25271848	1.32357568	1.38851287	0.03404289	0.00175978	0.00028411	
TcCLB.508163.330	1.42288729	2.54083657	3.47186196	0.15960604	2.2299E-06	2.2775E-10	
TcCLB.508165.130	2.19043112	3.33839154	4.2533704	1.0494E-13	1.6683E-34	3.4858E-49	
TcCLB.508165.310	1.97672251	2.41076114	2.91157486	2.9149E-06	3.0955E-11	6.9674E-16	
TcCLB.508241.50	1.4099315	1.40401103	1.39719192	1.5164E-06	2.3434E-07	4.7032E-07	
TcCLB.508389.90	1.74118991	2.39866402	3.1880139	0.00017514	2.4898E-11	6.5413E-19	

TcCLB.508539.150	1.78174802	2.81042915	3.75879176	0.06771829	4.7466E-05	1.5301E-07
TcCLB.508541.40	1.88140293	4.24398199	5.02997305	1.2676E-06	1.038E-36	2.121E-45
TcCLB.508609.10	1.44794907	1.35394884	1.4060665	0.02110382	0.02801039	0.01449433
TcCLB.509205.100	2.81870667	3.21340733	3.12726301	0.03443675	0.00453592	0.00618005
TcCLB.510263.30	1.17240556	1.36848984	1.20187968	0.26743209	0.00449	0.10400559
TcCLB.510281.20	1.84088179	3.0078708	3.47827164	7.5275E-10	4.0687E-34	3.955E-43
TcCLB.510307.10	1.66526121	1.91932455	3.52195239	0.01779351	0.00037029	2.404E-12
TcCLB.510565.150	1.64770797	1.87992282	1.99921327	1.0753E-09	1.0095E-16	1.3665E-19
TcCLB.511437.10	1.36842701	2.83309694	1.39157304	0.40695987	9.0173E-05	0.24981006
TcCLB.511701.10	1.38046945	2.42930632	2.42883695	0.57500139	0.02394052	0.02544528
IDs	FC 14vs7	FC 21vs7	FC 28vs7	pAdj 14vs7	pAdj 21vs7	pAdj 28vs7
TcCLB.507275.61	0.92643987	0.88284966	0.88105514	0.59653857	0.34381068	0.32449439
TcCLB.508163.400	5.88890867	3.52860164	4.17086948	NA	NA	0.3008088
TcCLB.508841.10	0.8761214	0.95483351	0.91829437	0.31944026	0.74866482	0.51647757
TcCLB.509123.10	1.00881113	0.91205043	2.44485891	0.98405762	0.81815618	0.00160076
TcCLB.509147.40	1.42133562	1.33851272	0.86724847	NA	0.71294948	0.86815422
TcCLB.510717.10	1.28479769	1.42314158	2.13019683	0.03546949	0.00150023	1.7476E-13
TcCLB.510717.20	1.30425513	1.42512525	2.20334601	0.00142722	8.2068E-06	3.0932E-26
TcCLB.511877.10	1.72474802	8.91277305	18.8003347	NA	NA	0.02491303
IDs	FC 14vs7	FC 21vs7	FC 28vs7	pAdj 14vs7	pAdj 21vs7	pAdj 28vs7
TcCLB.507485.30	1.72003647	1.72319506	2.00497236	0.02351267	0.00770962	0.0006722
TcCLB.507485.45	0.767743	0.60299554	0.57080176	0.17444366	0.00155005	0.0006524
TcCLB.507485.40	1.27098587	1.38557284	1.66467249	0.08744982	0.0046422	9.5958E-06
TcCLB.507673.60	2.19375874	2.78029626	2.62644251	0.10333159	0.00861092	0.0145983
TcCLB.507739.120	1.29335563	1.30765063	1.99361049	0.03788819	0.01028303	1.7353E-11
TcCLB.507485.150	0.48521226	5.85318302	10.565031	NA	0.01195218	0.00066728
TcCLB.509965.390	0.90393496	0.92744069	0.91294517	0.08093764	0.11495334	0.05906108
TcCLB.509965.394	0.93157584	1.15488537	1.38482501	0.62222422	0.14888743	0.00109542
